# The Role of Aqueous Solvation on the Intersystem Crossing of Nitrophenols

**DOI:** 10.1021/acs.jctc.3c01400

**Published:** 2024-04-12

**Authors:** Eva Vandaele, Momir Mališ, Sandra Luber

**Affiliations:** Department of Chemistry, University of Zürich, Winterthurerstrasse 190, 8057 Zürich, Switzerland

## Abstract

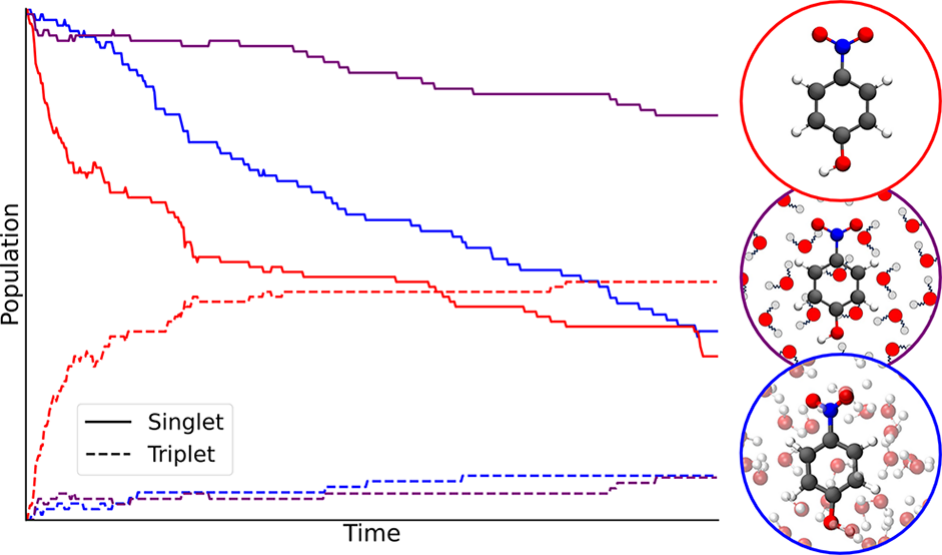

The photochemistry of nitrophenols is a source of smog as nitrous acid is formed from their photolysis. Nevertheless, computational studies of the photochemistry of these widespread toxic molecules are scarce. In this work, the initial photodeactivation of *ortho*-nitrophenol and *para*-nitrophenol is modeled, both in gas phase and in aqueous solution to simulate atmospheric and aerosol environments. A large number of excited states, six for *ortho*-nitrophenol and 11 for *para*-nitrophenol, have been included and were all populated during the decay. Moreover, periodic time-dependent density functional theory (TDDFT) is used for both the explicitly included solvent and the solute. A comparison to periodic QM/MM (TDDFT/MM), with electrostatic embedding, is made, showing notable differences between the decays of solvated nitrophenols simulated with QM/MM and full (TD)DFT. A reduced intersystem crossing in aqueous solution could be observed thanks to the surface hopping approach using explicit, periodic TDDFT solvation including spin–orbit couplings.

## 1 Introduction

Intersystem crossing (ISC) is a very important phenomenon in the photodeactivation of many chemical systems. The spin flip from a singlet to a triplet excitation character is in principle a forbidden transition, but spin–orbit coupling renders the radiationless process possible.^1,2^ Due to the low probability, ISC of standard organic molecules without heavy atoms is relatively slow, so that the overall decay after photoexcitation is often dominated by other pathways.^3^ Nevertheless, they are indispensable for many technological and biological applications. Organic light-emitting diodes (OLEDs) rely on phosphorescence.^4,5^ Other applications include bioimaging, chemical sensors, or data encryption.^6−8^ Nonradiative decay following ISC is targeted in photodynamic therapy (PDT) and photocatalysis.^9−11^ Whereas the former generates a reactive oxygen species after irradiation, the latter activates a compound by energy transfer, adduct formation, or noncovalent interactions with a substrate.^12,13^

The photoexcitation of nitrophenols has been investigated by several groups, as they exhibit fast ISC and are model systems to study intramolecular charge transfer or excited state intramolecular proton transfer (ESIPT) properties.^14−20^ Moreover, the photolysis of nitrophenol forms nitrous acid, which is a precursor for smog in the atmosphere.^21−25^ Already 50 years ago, health issues after photochemical smog exposure were ascribed to these compounds.^26^ Forest decline has been attributed to these phytotoxic, cytotoxic, and mutagenic compounds as well.^27−30^ Nitrophenols are ubiquitous in the atmosphere, especially in urban areas, and in the environment due to anthropogenic emission, e.g. from combustion processes, dye industry, or pesticide hydrolysis.^22,31,32^ Whereas *para*-nitrophenol (4-NP) occurs mainly as particles bound in clouds, a liquid phase enrichment of *ortho*-nitrophenol (2-NP), e.g. in fog and small cloud droplets, was measured.^33^ Even though 2-NP is more emitted, 4-NP is prevalent in the atmosphere.^30,34,35^ Both 2-NP and 4-NP are on the priority pollutant list of the United States Environmental Protection Agency.^36^

The ultrafast nonluminescent decay and ISC of all nitrophenol isomers have been confirmed with transient grating experiments.^37^ Triplet lifetimes are in the picoseconds range.^16,37^ In this study, the initial decays of both 2-NP and 4-NP will be investigated. The former has been more extensively examined due to the strong intramolecular hydrogen bond between the electron-donating and electron-withdrawing substituents and resulting environmental issues.^38−40^ After excitation to the first singlet excited state (S1), an intramolecular proton transfer from the phenol to the nitro group results in the nitronic acid tautomer.^16,22,41,42^ More specifically, the nitronic acid group will rotate out of plane, thereby decoupling the donor–acceptor groups, which leads to a conical intersection with the ground electronic state (S0). Alternatively, the torsion could precede the proton transfer, according to static potential energy surface (PES) reconstructions.^16^ The nitrous acid is formed by photolysis in the triplet manifold on a 0.5–1 ps time scale after excitation.^16,25,43^ The unstable isomer as well as ISC has been identified experimentally both in the gas phase and in solution.^16,25,41−45^

Nonadiabatic molecular dynamics (NAMD) simulations of 2-NP molecules revealed three key processes in the photoisomerization mechanism, with a prominent role of the first triplet excited state (T1).^15^ A direct hydrogen transfer, tunneling hydrogen transfer (estimated via a one-dimensional semiclassical method along the ESIPT coordinate^46^), and no hydrogen transfer process with time scales of 40 fs, 10 ps, and 300 fs and corresponding ratios of 13%, 36% and 48% in 280 surface hopping (SH) trajectories were found, respectively. The majority of the trajectories visited the second triplet excited state (T2) before further decay, thanks to an extensive ISC region with S1. On the experimental side, Alif et al. documented the phototransformation of 2-NP in various aqueous conditions.^47^ Photoproducts include catechol and nitrohydroquinone.^48^ More photoproducts were observed at shorter wavelengths. Stimulated emission was recorded in solution, as well as slower ISC dynamics.^16^ Interestingly, long-lived triplet states detected in the gas phase and in organic solvents are absent in aqueous solution.^17^ In contrast, UV-laser-induced photolysis was registered in water but not in methanol. According to the authors, it remains unclear whether this is due to absent ESIPT, hindered nitrous acid (HNO_2_) detachment or hydrogen backtransfer from the aci-tautomer after relaxation. However, a simultaneously published paper found minimal solvent effects between aqueous and isopropanol solutions of nitrophenols using UV/vis spectroscopy.^14^

The nonlinear optical properties are the main motivation to study 4-NP and its anion.^49,50^ Whereas research on 2-NP is mainly in the gas phase, studies of 4-NP focused on aqueous solution.^18,19,24,51,52^ Exploratory experiments found a displacement of the nitrate by nucleophilic photosubstition with hydroxyl from the aqueous solvent.^21^ Later research yielded nitrocatechol, hydroquinone, and other photoproducts depending on the conditions.^48,53^ Transient absorption spectra showed a pH-independent stimulated emission with a 150 fs decay time and vibrational relaxation on a 2.1 ps time scale and indications of deprotonation at longer delay times.^18^ Indications for a longer lived triplet were present for 4-NP but not for its corresponding anion.^54^ The solvatochromatic redshift has been well-established experimentally and reproduced theoretically using quantum mechanics/molecular mechanics (QM/MM) simulations.^19^ The aqueous solution and 4-NP conformational changes contribute equally to the redshift.^17^

Most NAMD studies in solutions are performed using QM/MM, whereby often only the solute is computed quantum mechanically.^55^ Nevertheless, large QM regions including many solvent molecules are necessary to converge a solvated system’s properties.^56,57^ Cluster models and QM/MM approaches including solvent molecules in the QM region have shown that the deactivation mechanisms and lifetimes depend on the quantum mechanical solvent molecules.^58−60^ As it is hard to predict the number and positioning of relevant solvent molecules beforehand, the errors generated by the omission of these molecules in the QM region can be avoided with a periodic, full ab initio model.^61,62^ Although SH has been commonly used for excited state dynamics studies, to the best of our knowledge, no SH approach using fully periodic time-dependent density functional theory (TDDFT) for a solvated system has been performed.

Even though 2-NP is regularly present in the liquid phase, the effect of aqueous solvation on the photodecay process and the reason for the experimentally observed slower ISC in solution are not clear. Moreover, the exact photodynamics of excited 4-NP is still unresolved, as previous research focused on the solvatochromatic shift and photoproducts. In this paper, the initial (250 fs) deexcitation of 2-NP in the gas phase, and in aqueous solution modeled with periodic QM/MM (TDDFT/MM) and explicit, periodic TDDFT solvation, respectively, will be described. Next, the initial decay of 4-NP in the gas phase and in aqueous solution modeled with QM/MM and explicit, periodic TDDFT solvation, respectively, will be revealed. A comparison between the QM/MM and full TDDFT models will be made to estimate the accuracy of the popular QM/MM approach for the photodecay of small organic molecules, whereby only the solute is modeled quantum mechanically. To the best of our knowledge, this is the first SH study using periodic explicit solvation with TDDFT as well as including the ISC of a solute in a periodic, explicit solvent comparing QM/MM and full (TD)DFT approaches for a large number of excited electronic states.

## 2 Methods

### 2.1 Theory

Although static calculations can reveal valuable information on the spectra, excited geometries, and critical points of the PES, dynamic calculations are indispensable to uncover the complex photodecay phenomena. The most commonly applied technique to model NAMD is SH.^63−65^ In this mixed quantum-classical, Born–Huang-based method, the molecular system evolves on an (excited) adiabatic PES, but it can change its state (hop) stochastically and instantaneously. More specifically, the nuclei are propagated classically on quantum mechanically computed electronic PESs. If no hop occurs, the nuclei are propagated classically based on the populated state’s gradients. After each nuclear displacement, the electronic structure is optimized, and the hopping probability is compared to a randomly generated number from a uniform distribution to determine if a change of electronic state will take place.

Different algorithms to compute the hopping probability have been developed. In this work, the Landau–Zener (LZ) approach has been applied including ISC.^66−69^ The transition probability for a hop (*P*^LZ^) between two nonidentical states (*i*, *j*) of the same multiplicity (X) with corresponding energies *E*_X*i*_ and *E*_X*j*_ is
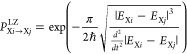
1with *ℏ* being the reduced Planck constant. The probability of ISC between singlet and triplet states is given by
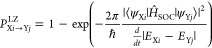
2The ⟨ψ_X*i*_|*Ĥ*_SOC_|ψ_Y*j*_⟩ term in the equation above refers to the spin–orbit coupling (SOC) between the wave functions describing states *i* (ψ_X*i*_) and *j* (ψ_Y*j*_).^70,71^ The LZ-SH algorithm was shown to perform equally well as Tully’s fewest switches (TFS) SH procedure for multiple excited electronic states of the same multiplicity,^66,72,73^ but ISC rates are underestimated as the ISC hops only occur at crossing points between singlet and triplet electronic state energies.^66^ While the LZ model was examined in various analytical three-state systems,^74,75^ the exact performance of trajectory branching at three-state conical intersections remains unknown.^66^ On the other hand, Granucci and co-workers have shown that the TFS-SH probabilities for ISC to a combined triplet electronic state are affected by the lack of phase continuity of the combined SOC term (see next paragraph) and that the LZ-SH performers better, even for larger SOC values.^69^ The reader should be aware that the internal conversion and ISC kinetics could be affected by the use of the LZ-SH algorithm. However, it does not influence the possible nonradiative deactivation mechanism for each individual trajectory.

Diverse approaches also exist to calculate the SOC terms. Linear-response TDDFT (LR-TDDFT) within the Tamm–Dancoff approximation was used in this work to compute the excitation energies.^76,77^ SOC terms are based on the one-electron term of the Breit–Pauli Hamiltonian where the effective charge approximation was chosen.^78,79^ In atomic units, the corresponding operator expression for a system with *N*_*e*_ electrons and *N*_*n*_ nuclei is
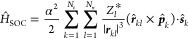
3with α being the fine structure constant, and *Z*_l_^*^ being the effective charge of nucleus *l*.^80^ The vector ***r***_*kl*_ represents the distance between electron *k* and nucleus *l*, while ***p̂***_*k*_ and ***ŝ***_*k*_ are the electron’s momentum and spin angular momentum operators, respectively. The final SOC value is obtained by the root sum squared of the three triplet projection terms (*M* = −1, 0, and +1) to combine the three energy degenerate triplet electronic states into one effective triplet state^81,82^
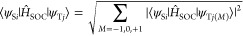
4

If the hopping probability exceeds the random number, the system changes state, and all velocities are rescaled in order to conserve the total energy.^83,84^ In the case insufficient kinetic energy is available for an upward hop, the transition is rejected. As the nonadiabatic hops are stochastic, a collection of independent trajectories with various initial geometries and velocities are evolved to properly sample the photodeexcitation.

### 2.2 Computational Settings

All DFT, LR-TDDFT, and QM/MM calculations were performed using a modified version of the CP2K program version 2023.1 at the B3LYP/DZVP-GTH level of theory with the mixed Gaussian and plane wave DFT method and Grimme’s D3 dispersion correction without damping.^85−89^ A good agreement between 2-NP optimized geometries using the TDDFT and state-averaged complete active space self-consistent field (SA-CASSCF) methods was previously established,^16^ while B3LYP proved to be a rather accurate functional for computing 2-NP ground state properties and excitation energies.^90^ In addition, the optical properties of 4-NP computed at the B3LYP/6-31+G(d,p) level of theory were previously shown to be comparable to second-order Mo̷ller-Plesset perturbation theory (MP2) and experimental results.^49^ The auxiliary density matrix method (ADMM) was applied to reduce the computational cost with an exact exchange cutoff radius of 5 Å.^91,92^ The Tamm–Dancoff approximation was applied, which suffers less from the triplet instability problem.^93^ The long-range corrected CAM-B3LYP functional was employed for excited electronic energy calculations of some selected trajectory points.^94^ The LZ trajectories were propagated by a modified version of the Zagreb surface hopping code including ISC.^95^ An interface script tailored to LR-TDDFT output files from CP2K was written in Python to couple both programs and to compute the LR-TDDFT SOC terms. The conical intersections were optimized using the CIOpt program.^96^ All figures of molecular orbitals used an isosurface value of 0.05 au. The theoretical absorption spectra obtained from the first ten singlet excitation energies and corresponding oscillator strengths of all sampled initial configurations were broadened using a Lorentzian function with a half-width at a half-maximum height of 10 nm.

Initial conformations and velocities for the gas phase NAMD calculations were sampled from the thermalized Wigner distribution at 300 K.^97,98^ The molecules were centered in a nonperiodic box with a cell length of 15 Å. The aqueous systems were prepared by centering the nitrophenol molecules in a water box pre-equilibrated at ambient conditions.^99,100^ The boxes had an initial cell length of 12.4 Å and contained 50 water molecules after the addition of the solute. This is sufficient to include the first solvation shell and the majority of the second solvation shell. DFT molecular dynamics trajectories of 5 ps at the BLYP/TZV2P-GTH level of theory were run in the isotropic isothermal–isobaric (NPT) ensemble at 300 K and 1 bar with a time step of 0.5 fs. The temperature, using the Nose–Hoover chains thermostat, and pressure were equilibrated with a time constant of 50 and 200 fs for the thermostat and barostat, respectively.^101,102^ Averaged cell lengths of 11.45 and 11.52 Å were found for solvated 2-NP and 4-NP, respectively (averaged after discarding the first 2 ps), during the NPT simulations. Using these cell lengths, the systems were further evolved in the canonical ensemble (NVT) at 300 K using a 200 fs time constant for the Nose–Hoover chains thermostat. Starting geometries and corresponding velocities for the NAMD simulations were sampled from 50 ps of NVT trajectories.

An analogous strategy was followed to generate the initial conditions for the periodic QM/MM trajectories. Whereas the solute was treated quantum mechanically by means of (TD)DFT, an MM model was applied for all solvent molecules. Force field and electrostatic embedding parameters were supplied by the general Amber force field (GAFF) in combination with the AM1-BCC charge method and the TIP3P water model as implemented in the Ambertools program.^103−106^ A 10 Å cutoff radius for nonbonded interactions, the fast Gaussian expansion of the electrostatic potential, and additional Lennard–Jones terms between the solvent and solute were defined.^107^ The full DFT NPT-equilibrated conformations used as input for the subsequent DFT NVT trajectories were further relaxed in the NPT ensemble by using QM/MM (DFT/MM) for over 15 ps. Notwithstanding otherwise identical computational settings, cell lengths of 11.69 and 11.80 Å were obtained for solvated 2-NP and 4-NP, respectively. The initial QM/MM phase space points for the NAMD trajectories were sampled from 50 ps of NVT periodic QM/MM simulations.

For each setup, 100 LZ SH trajectories were propagated for 250 fs with a 0.5 fs time step. All 2-NP trajectories were initiated from the S1 state. In the case of 4-NP, the trajectories were initiated from different singlet excited states, as interchanges of characters and brightness were frequent as compared to 2-NP. All 4-NP trajectories were initiated from the brightest π → π* state. This sampling of states also corresponded to the highest transition probability by the method given in ref.^108^ in 99% of the cases (due to mixing of the state characters). For 2-NP, two singlet and four triplet excited states were computed each step, while for 4-NP, four singlet and seven triplet excited states were computed each step. SOC terms are solely based on the solute. The implementation of the LR-TDDFT SOC terms was verified by comparison to the ORCA program.^109,110^ Forward–backward hops between the same states taking place within 5 fs were omitted for the population and hopping/ISC analyses but not corrected for during the LZ SH trajectories.

## 3 Results and Discussion

### 3.1 *ortho*-Nitrophenol

#### 3.1.1 Gas Phase

**Figure 1:**
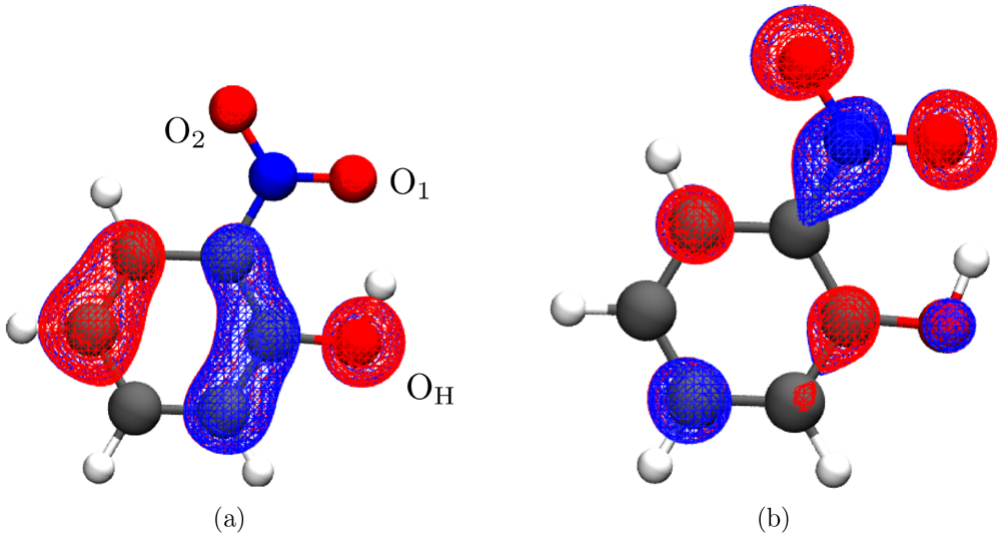
HOMO (a) and LUMO (b) of 2-NP.

The structure of 2-NP, and more specifically its intramolecular hydrogen bond, already interested chemists over 50 years ago.^38,111^ Excitation of 2-NP at the minimal energy ground state optimized geometry populates the S1 state at 3.54 eV, which has a π → π* character.^16^ At the B3LYP/DZVP-GTH level of theory, this corresponds to a highest occupied molecular orbital (HOMO) to lowest unoccupied molecular orbital (LUMO) transition, shown in Figure 1. The S1 excitation energy is 0.8 eV lower as compared to the SA-CASSCF results of Xu et al.^20^ and 0.5 eV lower as compared to the complete active-space second-order perturbation theory (CASPT2) results of Nitta et al.^25^ An experimental absorption maximum at 3.65 eV (340 nm) was found, which corresponds well to the absorption spectrum obtained in this work (SI Figure S1).^112^ Experimentally, an excitation wavelength for the S1 state of 355 nm has been used.^41,44^ The second singlet excited state (S2) is close at 3.86 eV but not a bright state. The *n* → π* state excites the nitro and hydroxyl oxygens’ free electron pairs to the LUMO (SI Table S2 and Figure S2). At the minimal energy ground state optimized geometry four triplet states, three π → π* and one *n* → π* transition, have a lower vertical excitation energy than the S1 and S2 states. The first triplet excited state (T1) has the same character as S1 but is 0.82 eV lower in energy, in line with the results of Ernst et al.^16^ The excitation energies for all states averaged over all initial conformations are given in SI Table S2.

Geometry optimizations of the S0, S1, and T1 states revealed three distinct structures. While the S0 minimum energy geometry is flat, the two nitro oxygens are out-of-plane in a pyramidalized fashion in the S1 optimized geometry. The T1 conformation features a proton transfer from the hydroxyl to the nitro group. This is in line with the intramolecular charge-transfer (CT) character of the π → π* excitation (Figure 1), as some electronic density is transferred from the benzene and hydroxyl groups to the nitro group. The hydroxyl proton can then follow the shift in the electronic density. An analogue T1 minimum was found at the SA-CASSCF(10,10)/6-31G(d,p) level of theory, while the nitro and hydroxyl groups remain almost planar in the S1 minimum at the same level of theory.^20^ The most important internal coordinates are given in Table 1. The S0 and T1 structures match those of Ernst et al., although their S1 minimum optimization at the BP86/def2-SV(P) TDDFT level failed. The difference between the N–O bond lengths and the O_1_–H distance in the S0_*opt*_ conformation agrees with values found in an electron diffraction analysis,^39^ and all internal coordinates compare well to X-ray 2-NP crystal studies^113^ and those of an S0 geometry optimized at the SA-CASSCF(10,10)/6-31G(d,p) level of theory.^20^

**Table 1:** Comparison of the 2-NP Structural Parameters at the S0, S1, and T1 Optimized Geometries and at the CI_S0/S1_ Conformation^a^

2-NP	S0_*opt*_	ref.^113^	ref.^20^	S1_*opt*_	ref.^20^	T1_*opt*_	ref.^20^	CI_S0/S1_
C–O_H_ [Å]	1.35	1.34	1.34	1.33	1.34	1.28	1.23	1.32
O_H_–H [Å]	0.99	0.80	0.95	1.05	0.95	1.62	2.41	1.11
C–N [Å]	1.45	1.46	1.45	1.49	1.39	1.43	1.43	1.65
N–O_1_ [Å]	1.26	1.23	1.21	1.31	1.23	1.39	1.37	1.40
N–O_2_ [Å]	1.23	1.20	1.20	1.26	1.43	1.25	1.25	1.30
O_1_–H [Å]	1.72	1.91	1.88	1.52	1.90	1.01	0.95	1.50
ONO [deg]	122	123	123	124	114	118	114	112
CNO_1_ [deg]	118	118	118	115	129	120	113	98
COH [deg]	107	109	111	103	111	-	-	98
CCNO_1_ [deg]	1	-	0	–9	–1	1	61	–41
CCNO_2_ [deg]	–179	-	–180	–170	–177	–179	–162	–157
CCOH [deg]	0	-	0	1.7	0	-	-	–1.4

a O_H_ indicates the hydroxyl O atom, O_1_ indicates the nitro O atom forming a hydrogen bond with the hydroxyl group, and O_2_ indicates the nitro O atom pointing away from the hydroxyl group.

In addition, a conical intersection between the S0 and S1 states (CI_S0/S1_) was optimized. This geometry does not correspond to a minimum energy CI as the penalty function method was used for the optimization,^114^ and one should keep in mind that restricted LR-TDDFT has a wrong dimensionality at CIs,^115−117^ but it gives a first indication of the deactivation mechanism. The geometry is characterized by an out-of-plane, pyramidalized nitro group, increased C–N and hydroxyl O–H bond lengths, and deformed ring structure. This structure is 1.03 eV higher in energy as compared to the optimized S1 minimum geometry.

Even though a Zhu-Nakamura SA-CASSCF(10,10)/6-31G(d,p) NAMD study of 2-NP in the gas phase has previously been performed,^15^ a set of 100 2-NP trajectories in vacuum was propagated in this work in order to disclose the solvation effects using a consistent level of theory. Whereas only three excited states were included by Xu et al.,^15^ two singlet and four triplet excited states were computed in this study. Nevertheless, only the initial deactivation process after charge-transfer excitation has been examined.

**Figure 2:**
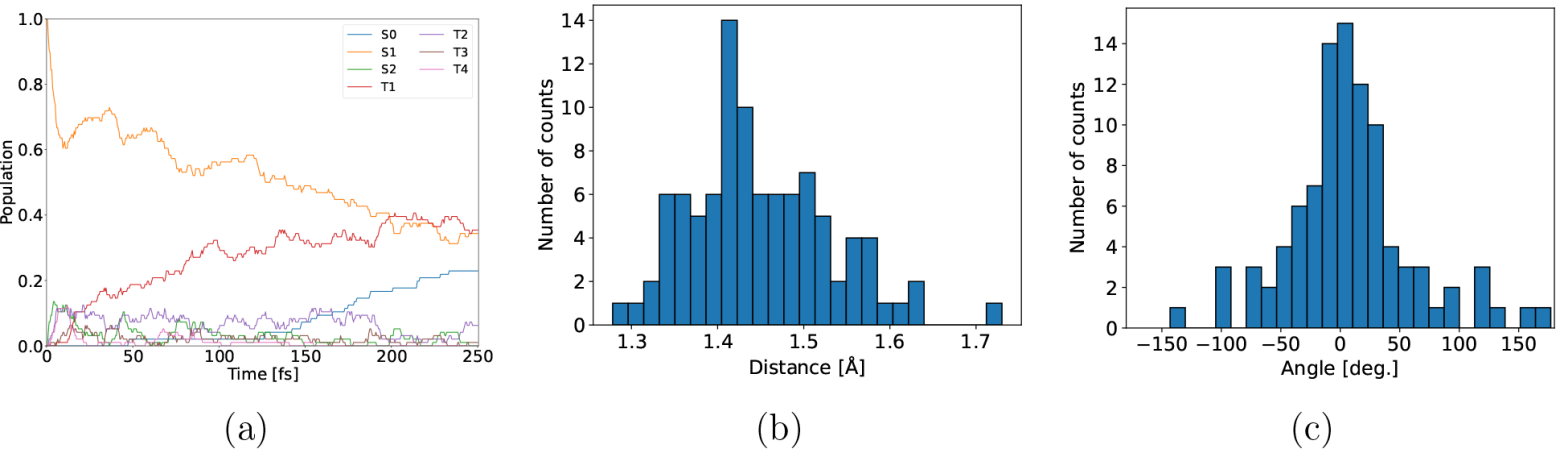
Population evolution of 2-NP in the gas phase after excitation to the S1 state (a) and histograms of the C–N distance (b) and CCNO_1_ dihedral angles of all conformations after 250 fs.

**Figure 3:**
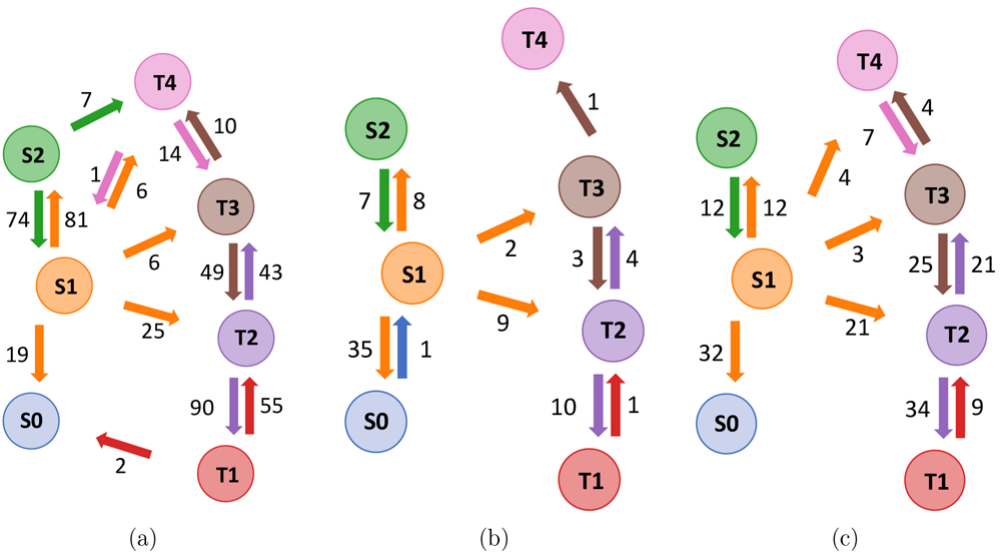
State diagrams summarizing hops and ISCs observed during the 250 fs 2-NP trajectories in the gas phase (a), in full TDDFT aqueous solution (b), and in aqueous solution modeled with QM/MM (c). The numbers next to the arrows indicate the absolute number of respective hops counted during the trajectories.

All NAMD trajectories were initiated from the S1 state, which mostly had π → π* character. However, in 11% of the initial conformations sampled from the Wigner distribution, the S1 and S2 characters were reversed, whereby the S1 state corresponded to a *n* → π* (HOMO–2 → LUMO) character, and the close-lying S2 state was a π → π* excitation. An initial steep decrease in the S1 population is evident in Figure 2a. The population is mainly transferred to the S2 and T4 states, which are energetically close to the S1 state. Of all of the trajectories in the S2 state, 92% transfer back to the S1 state, with the remaining 8% undergoing ISC to the T4 state (Figure 3a). This is reflected in a regrowth of the S1 population. Within 250 fs, only 20% of the trajectories display internal conversion from the S1 to S0 state, and except for two decays after 47 fs, all take place in the second half of the simulation time. The geometries at the hopping points are characterized by an out-of-plane, pyradimalized nitro group, ring deformations and frequently show ESIPT. This is due to the intramolecular CT character of the populated π → π* S1 state (Figure 1), which weakens the conjugation within the benzene ring and adjacent groups and increases the electronic density on the nitro group. All these geometric deformations are reflected in an increase of the S0 energy at the hopping points. Nevertheless, the various hopping geometries are quite diverse, indicating that many points along the CI seam are accessible. The other part of the S0/S1 seam corresponds to the *n* → π* (HOMO–2 → LUMO, see SI Figure S2) excitation type localized on the nitro group. This character makes up 63% of the trajectories remaining in the S1 electronic state at the end of the 250 fs simulation time, with only a few deactivated to the S0 state. The remaining population in the S1 state at the end of the simulation time has π → π* (HOMO → LUMO, see Figure 1) excitation character. In addition, two trajectories deactivated through ISC from the S1 to T2 state followed by internal conversion to the T1 state and ISC back to the ground state. After 250 fs, 43% is in a triplet state, one of which in the T3 state, six in the T2 state, and all others in the T1 state. The T1 electronic state is again dominated by the π → π* character (see Figure 1). The trajectory population evolves around the T1 minimum until the end of the simulation time. Several trajectories, except two, exhibit crossings between T1 and S0 PESs but do not deactivate to the S0 state. The trajectories remaining in the T2 electronic state are occupying the *n* → π* character localized on the nitro group, while the one in the T3 state demonstrates a benzene π → π* excitation. No jumps from the S1 to T1 state were recorded, although Xu et al. reported these as *“rare cases”*. This is expected as the T1 state mostly has the same character as the S1 state and remains lower in energy than the S1 PES, while the LZ-SH algorithm works only when the two PESs cross. The majority of the ISCs, 68%, happened between the S1 and T2 states (Figure 3a). Only one reverse ISC occurred, 11 fs after the 2-NP hopped from the S1 to T4 state. Geometrical deformations are observed at ISC points as well but are much less pronounced as compared to the internal conversion to the ground state. Last, frequent internal conversion between the energetically close triplet states is perceived, whereby the LZ-SH algorithm is still valid on average.^66,72,73^ In contrast to the TFS-SH algorithm, where the electronic population evolves as the trajectory is propagated, the LZ-SH algorithm does not consider the details of electronic population evolution. Hence, the ISC might be underestimated with the LZ-SH algorithm, as SHs are only considered at PESs crossing points and not by the electronic population change, as with the TSF-SH procedure. The SOC terms, for example between T1 and S0 states for trajectories in T1, are on average 60 ± 40 cm^–1^, occasionally ranging up to 600 cm^–1^. Similar issues exist in other states.

ESIPT is a fast process. Overall, 31% of the 2-NP molecules converted into the transient aci-nitro tautomer (SI Figure S3a), while ESIPT was taking place in 6 additional trajectories at 250 fs. Another 26% of the systems exhibited proton transfer during the dynamics, followed by a backtransfer. The proton transfer is present in all states as it is nearly barrierless^16^ and is facilitated by the intramolecular CT character of the π → π* (see Figure 1) state. Nitronic acid in the ground state always has an out-of-plane rotation of the aci-nitro group. Figures 2b and 2c summarize the distributions of the C–N distances and CCNO_1_ torsional angles at 250 fs, respectively. No nitrous acid has been created yet in the short time scale, but elongated C–N bonds are present. The N–O bonds are on average elongated in the excited states, singlet as well as triplet.

#### 3.1.2 Aqueous Solution

**Figure 4:**
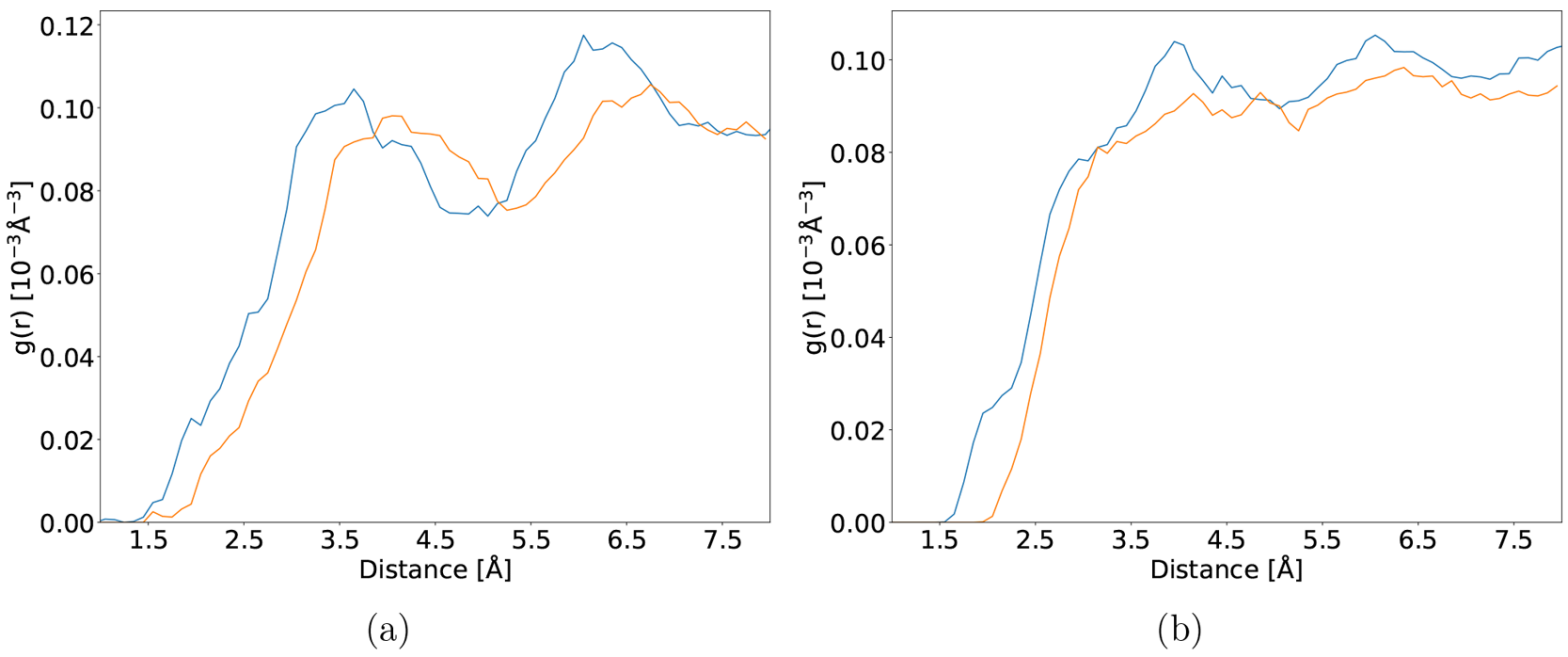
Radial distribution functions of the 2-NP hydroxyl hydrogen (a) and O_1_ nitro oxygen atom pointing toward the hydroxyl group (b) with the oxygen and hydrogen atoms of the solvent respectively made from ground state MD trajectories using QM/MM (orange) and full DFT (blue) calculations.

In aqueous solution, a bathochromic shift is observed in the maxima of the absorption spectra analogous to experimentally measured absorption spectra (SI Figure S1).^16^ Nevertheless, both spectra computed with QM/MM and full TDDFT are red-shifted as compared to an experimentally measured absorption spectrum in aqueous solution.^17^ The difference between QM/MM and full TDDFT solvation is small, with an additional 9 nm redshift for the latter. A larger difference is observed in the geometries. As noted in subsection 2.2, equilibration of the periodic simulation boxes engendered a cell length difference of 0.23 Å. This is reflected in the radial distribution functions (RDFs, Figure 4). In the ground state, the RDF of the hydroxyl hydrogen from the QM/MM trajectory reveals a bigger offset and shifted maximum of the first hydration shell. The differences between QM/MM and full DFT solvation are also present for the oxygens of the nitro-group. The RDF of the oxygen atom, which is hydrogen bonded to the hydroxyl group, displays slightly notable solvation shell features, whereas the RDF from QM/MM builds no hydrogen-bonded network with the surrounding aqueous solvent. Moreover, while the average distance between the oxygen atom and the closest hydrogen atom from the solvent is 2.31 Å with DFT, a value of 2.63 Å is obtained with QM/MM. Similar to the S0 optimized geometry, a difference between the N–O bond lengths of −0.02 and −0.03 Å is measured in full DFT solvation (average bond lengths of 1.28 and 1.26 Å) and QM/MM (average bond lengths of 1.28 and 1.25 Å), respectively.

The Franck–Condon excitation energies for the S1 state of 2-NP in aqueous solution are slightly higher with QM/MM as compared to full TDDFT solvation, with average excitation energies of 3.23 ± 0.16 eV and 3.03 ± 0.23 eV, respectively. In fact, all average QM/MM excitation energies are lower as compared to those of the gas phase, while the full TDDFT solvation features the lowest average excitation energies (SI Table S2). In both cases, 4% of the Franck–Condon S1 excitations do not correspond to the brightest state. With QM/MM, all 4% have character different from that of the brighter π → π* S2 state. In contrast, with full TDDFT 2% of the conformations indicate predominant π → π* contributions for both S1 and S2 states, which are energetically close, with S2 being brighter than S1.

**Figure 5:**
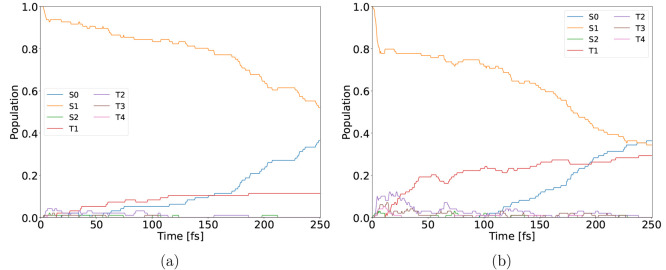
Population evolution of 2-NP in aqueous solution after excitation to the S1 state calculated using full TDDFT (a) and QM/MM (TDDFT/MM) (b).

The population evolutions during the photodecay processes shown in Figure 5 are clearly distinct. Whereas in the gas phase a fast initial reduction of the S1 population was conspicuous, it is much less pronounced in aqueous solution (SI Figure S4). ISCs take place, albeit reduced, but deactivation to the ground state takes precedence. Hence, a shorter excited state lifetime is to be expected in aqueous solution. Using QM/MM the population evolution features a combination of the gas phase and full TDDFT solvated results.

If the aqueous solvent is computed at the same level of theory as the solute, merely 11% of the trajectories are involved in ISC, mostly from the S1 state to the T2 state but also from the S1 to the T3 state, while no ISCs via the S2 state are present (Figure 3b). No ISC from the triplet electronic state to the S0 state is observed. The decay is in line with the experimental observation of reduced and slower ISC in solution.^16,17^ At the end of the 250 fs simulation time, 37% of trajectories reached the S0 state after internal conversion from the S1 state, from which one-sixth deactivated from S2 over S1 to S0. The remaining 52% and 11% of trajectories stayed in the S1 and T1 states, respectively, both being of the π → π* (see Figure 1) character. ESIPT was observed in 7% of trajectories, with 5% as aci-nitro tautomers and 2% as conformations with the hydrogen atom in between the nitro and hydroxyl groups at exactly 250 fs, but only in the T1 states. At that point, the trajectory with the longest C–N bond has a 1.62 Å bond length, so no nitrous acid creation is observed. Nevertheless, a variety of CCNO_1_ dihedral angle values is adopted.

As opposed to that in the gas phase, ESIPT is not (nearly) barrierless in aqueous solution (SI Figure S3a). Despite the equal intramolecular π → π* CT character of the S1 and T1 states, the barrier seems larger for the singlet state compared to the triplet state. The hydroxyl group is never deprotonated at the ISC geometries, which are structurally close to the S1-optimized conformation. The conformations at the hopping points between the S1 and S0 states are much more deformed, often, but not exclusively, featuring an out-of-plane nitro group and sometimes elongated hydroxyl bonds.

The trajectories that first hop from the S1 to S2 state (6%) changed from a π → π* character to an intermolecular CT state. A natural transition orbital analysis showed that the latter state is a CT with excitation from the water *n* MO to the π* MO localized on the nitro group. Once in the intermolecular CT state, one of the O–H bonds of the water molecule involved in the excitation significantly starts to stretch, causing the S0 and S1 state energies to suddenly rise. The trajectory population first switches from the S2 to S1 state at their trivial crossing point and continues having an intermolecular CT character along the S1 state until the CI with the S0 state has been reached. After the first hop to the S2 state with intermolecular CT character, the trajectory deactivates to the ground electronic state in a few tens of fs. Recalculation of these S0/S1 CI points with the CAM-B3LYP functional confirms the same intermolecular CT character of the S1 state as obtained with the B3LYP functional.

With QM/MM, 21% of the trajectories participate in ultrafast ISC, analogous to gas phase results. However, the sharp initial decline of the S1 population is restricted as compared with gas phase results. Hops between the S1 and S2 states occur more frequently as compared to full TDDFT results (Figure 3c), but no system spends extended periods of time on the S2 PES. Within 250 fs, 30% exhibit ISC between the S1 and T2 states and to a lesser extent between the S1 and T3 or T4 states. Last, 34% of the excited systems have reached the S0 state within the simulation time, exclusively after a hop from the S1 PES. The characters of electronic states are the same as the corresponding gas phase electronic states. Figure S5 compares the cumulative singlet and triplet electronic state population evolutions for 2-NP trajectories between the gas phase and full TDDFT and QM/MM solvations.

The distribution of the C–N distances and CCNO_1_ dihedral angles after 250 fs is analogous to full TDDFT results, with only a 10° smaller standard deviation for the latter. Nonetheless, ESIPT is more frequent athwart full TDDFT, with 26% nitronic acid and 7% in the process of tautomerization after 250 fs. ESIPT happens both on the S1 and T1 PESs and clearly facilitates the ISC.

### 3.2 *para*-Nitrophenol

#### 3.2.1 Gas Phase

**Figure 6:**
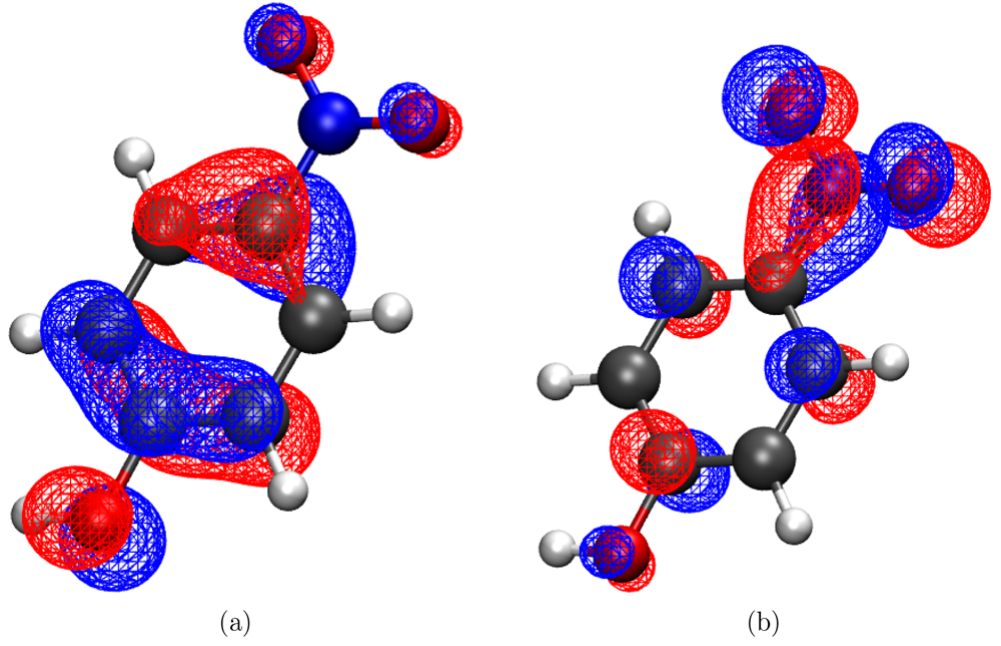
HOMO (a) and LUMO (b) of 4-NP.

The energetically lowest spectroscopically bright 4-NP excited state corresponds to a π → π* (HOMO → LUMO at the B3LYP/DZVP-GTH level of theory, see Figure 6) electronic excitation, characterized by an increase of the electric dipole moment.^19^ This is similar to the π → π* intramolecular CT character of 2-NP, whereby an electronic density shift from the hydroxyl toward the nitro group is observed. At the S0 minimum energy geometry, this state is the third singlet excited state (S3), proceeded by a *n* → π* (HOMO–2 → LUMO) S1 and a *n* → π* (HOMO–4 → LUMO) S2 excitation. The Franck–Condon S3 excitation energy at the S0 optimized geometry amounts to 4.46 eV. However, the theoretically computed absorption spectrum generated from 150 4-NP geometries randomly sampled from the Wigner distribution corrects the lowest π → π* excitation energy to a value of 4.55 eV, slightly below the experimentally measured maximum (at 4.71 eV) in the gas phase.^50^ The S2 and S4 excitation energies are relatively close, within 0.23 eV. The S4 state is a π → π* (HOMO–1 → LUMO) excitation at the S0 minimum energy geometry. Seven triplet states with a Franck–Condon excitation energy lower than the S3 excitation energy at the S0 minimum energy geometry were all included in the SH as well (SI Table S3 and Figure S6), as there is no previous 4-NP NAMD study to indicate the number of populated excited states. Bond lengths and angles at the S0 optimized geometry compare well to experimental values (SI Table S4). The optimized T1 minimum energy geometry and optimized CI_S0/S1_ feature an out-of-plane nitro group (SI Table S4), but the CI_S0/S1_ also has an out-of-plane hydroxyl group. Whereas the O–N–O angle and C–N bond length are reduced in the excited states, the N–O bond lengths are increased. While the S1 and T1 minima are 2.95 and 2.46 eV above the S0 minimum energy, respectively, the optimized CI_S0/S1_ is 3.61 eV higher in energy as compared to the S0 minimum.

Considering the energetically close-lying singlet excited states at the S0_*opt*_ structure, small geometric deviations can cause permutations of the state characters. From the propagated trajectories, the brightest state proved to be S3 for merely 41% of the initial conformations, while it was S1, S2, and S4 for 6%, 16%, and 37%, respectively. The brightest state always had mainly π → π* (HOMO → LUMO) excitation character.

**Figure 7:**
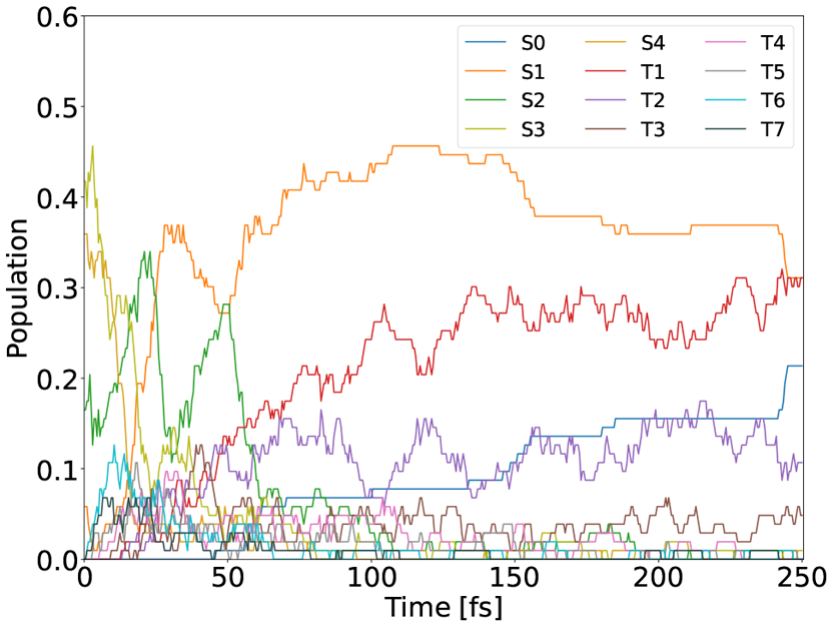
Population evolution of 4-NP in the gas phase.

The distribution of the initial states is reflected in the population evolution graph (Figure 7). Naturally, the S4 and S3 populations show sharp initial decreases, accompanied by a clear increase in the S2, S1, and T6 populations. Toward the end of the 250 fs simulation period, the S0, S1, and T1 states are the most populous. A numerical overview is given in SI Table S5. In terms of excited electronic state characters, the trajectories start with a π → π* character, which is mixed with the two energetically lower *n* → π* characters, and evolve toward a pure *n* → π* by transitioning to the energetically lowest singlet excited electronic states. All the S1 states, before their SH to S0, and energetically lowest triplet states at the end of the simulation time are of *n* → π* character, which is completely localized on the nitro group. All states included in the simulations participated in the decay, and ISC is feasible from all singlet excited states. In total, triplet states were involved in the decay of 58% of the trajectories. ISC trajectories in the S4 state transitioned mainly to the T7 state, but a few fast hops to the T6 state occurred as well (SI Figure S7). ISC from the S3 state mainly contributed to the T6 population increase with transitions to the T7 and T5 states observed to a lesser extent. In addition, transitions to the T6 state were preferred from the S2 state, as well, with minor contributions to the T5 and T4 states. A single transition from the S2 to the T7 state was noted. Finally, only two transitions from the S1 to T5 state and two from the S1 to the T3 state were registered. All deactivations to the ground state started from the S1 state with a first hop after 45 fs. It should be noted that a number of trajectories display frequent internal conversion processes between states of equal multiplicities (at the beginning between closely lying singlet excited states and similarly between triplet states after the ISC event), which leads to noticeable oscillations in individual state populations as shown in Figure 7. While such frequent SH events might question the validity of the used LZ-SH algorithm, particularly when three electronic states of the same multiplicity come close together in energy, cumulative populations of each multiplicity display monotonic changes without any noticeable oscillations (SI Figure S8) and are in line with previously reported LZ population evolution behaviors.^66,72,73^

**Figure 8:**
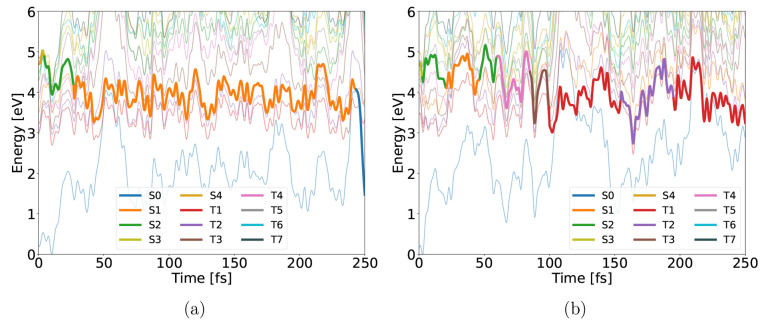
Energy evolution of two representative 4-NP trajectories in the gas phase, showing internal conversion to the S0 state (a) and ISC between the S2 and T4 states (b). The occupied state is shown with an increased line width.

After a decay time of 250 fs, one *para*-nitrophenolate anion was formed. The dissociation took place in the T1 state. On average, the O–H bond lengths are extended in the excited states as compared to the S0_*opt*_ geometry. In the first 25 fs after excitation, the bond lengths of the parallel C_H_–C_H_ bonds increase by ±0.07 Å, while the N–O bond lengths also increase by at least 0.1 Å. Overall, the excitation energy is manifested in large variations of all bond lengths (SI Figure S9). Consequently, all angles and dihedrals also show rather large deviations from the S0_*opt*_ values to accommodate for the changing bond lengths while keeping the structural integrity of the molecule. This induces a great increase in the S0 energy, up to the point where an internal conversion from the S1 state is possible, as shown in Figure 8a. A representative trajectory including ISC is shown in Figure 8b. The system first decays from the S3 to S1 state, followed by a hop back to the S2 state and ISC to the T4 state, which deactivates to the T1 state. Four trajectories still feature a C–N bond length elongation of over 0.25 Å as compared to the S0_*opt*_ geometry at the end of the simulation time after having fully decayed to the S0 state in less than 250 fs. However, the average C–N bond length decreases with time. A full out-of-plane rotation of the nitro group or the hydroxyl group is very rare. For example, no twisted nitro groups with respect to the phenol ring are present at 250 fs, as reflected by a standard deviation of only 19° for the CCNO dihedral angle distribution. No systematic alterations in the CCNO dihedral angles at the hopping points are evident either. Smaller O–N–O angles are noticed in the S1, T1, and T2 states. Consequently, the C–N–O angles are generally increased in these states.

#### 3.2.2 Aqueous Solution

**Figure 9:**
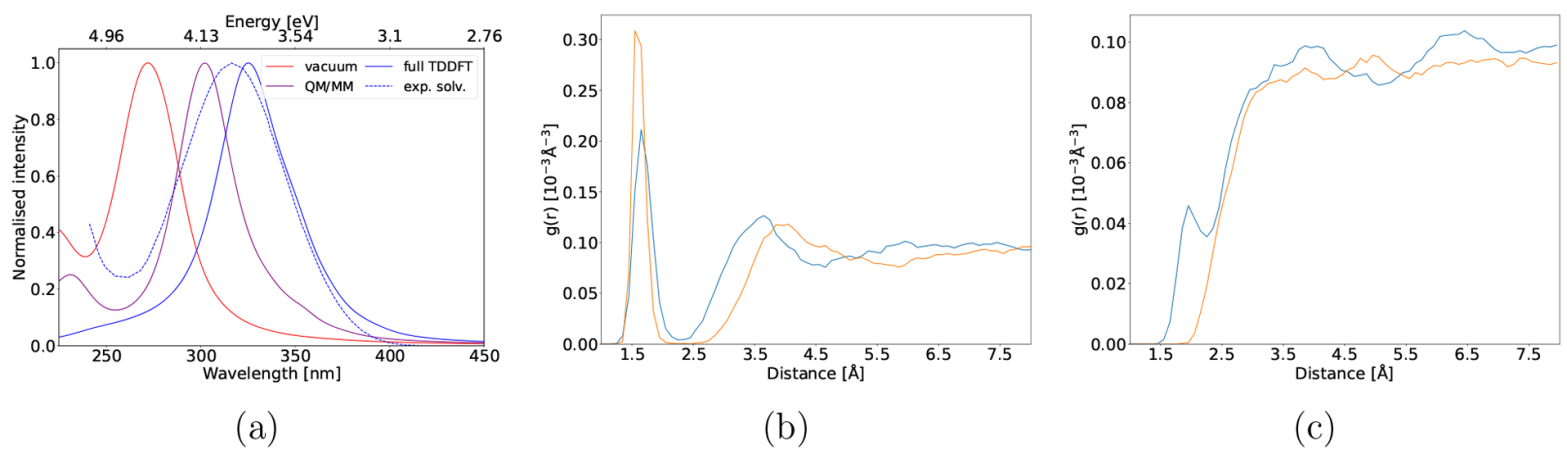
(a) The TDDFT absorption spectrum of 4-NP in the gas phase (red) and in aqueous solution modeled with QM/MM (purple) and full TDDFT (blue) generated from the first ten excitations of 150 snapshots. The dashed curve corresponds to an experimentally measured absorption spectrum of 4-NP in aqueous solution reproduced from ref (17). Radial distribution functions of the hydroxyl hydrogen with respect to the solvent oxygen atoms (b) and both nitro oxygens with respect to the solvent hydrogen atoms (c) made from 5000 snapshots uniformly sampled from 50 ps ground state MD trajectories by using QM/MM (orange) and full DFT (blue) calculations.

Most previous 4-NP studies were performed in solution and elaborated on the solvatochromic shift and pH dependence.^17−19^ Experimentally measured absorption spectra in aqueous solution located a maximum at 3.88 eV,^52^ 3.90 eV,^50^ or 3.91 eV.^17,51^Figure 9a summarizes the 4-NP absorption spectra calculated in this work. Whereas the absorption peaks at 3.81 eV in full TDDFT solvation, a reduced redshift as compared to the gas phase is obtained with QM/MM (maximum at 4.1 eV). A previous CASPT2(12,10)/MM study reported redshifts of 0.71 and 0.94 eV as compared to a gas phase structure with the polarizable continuum and free-energy gradient method optimizations, respectively.^19^ They suggested that nearly half of the solvatochromic shift arises from the solvent effect on the solute structure. Nevertheless, only minimal geometrical differences between the periodic QM/MM and full, periodic DFT S0 NVT trajectories were observed in this work (SI Figure S10). The QM/MM absorption maximum does not change if only geometries sampled from the last 10 ps of the 50 ps ground state MD trajectory are used to generate the spectrum compared to randomly sampled geometries from the full trajectory. Although the 4-NP radial distribution functions (9b and 9c) are much more aligned as compared to 2-NP, the QM/MM generated curve in Figure 9c is almost featureless. Moreover, the small peak indicating hydrogen bonding with the solvent present between 1.5 and 2.0 Å in the full DFT radial distribution function is missing. Using full DFT solvation, both oxygen atoms of the nitro group engage in hydrogen bonding with various surrounding solvent molecules during the trajectory. The solvent molecules forming the hydrogen bonds regularly interchange, leaving intermediate periods during which no hydrogen bonding is observed, which explains the small peak height in the radial distribution function in Figure 9c. Both nitro group oxygen atoms are rarely engaged in hydrogen bonding at the same time. The average distance between the closest oxygen atom from the solvent and hydroxyl hydrogen atom of the solute is 1.73 and 1.64 Å with full DFT and QM/MM solvation, respectively, whereas the average distance between the closest hydrogen atom from the solvent and nitro oxygen atoms of the solute is 0.42 Å further away with QM/MM as compared to full DFT solvation.

**Figure 10:**
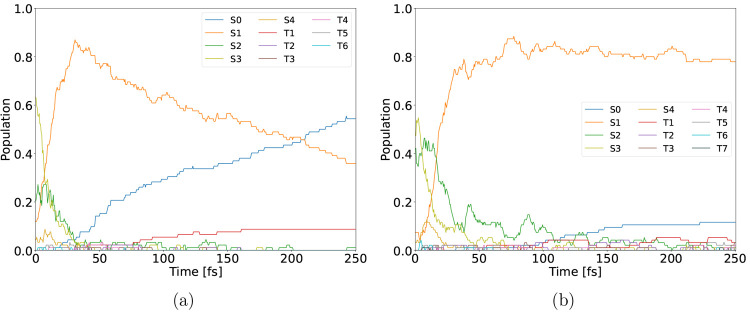
Population evolution of 4-NP in aqueous solvation modeled with full TDDFT (a) and QM/MM (b).

In line with the gas phase SH simulations, the 4-NP QM/MM trajectories were initiated from the state with the highest TDDFT oscillator strength, having a predominant π → π* (HOMO → LUMO) character, which was the S1, S2, S3, and S4 states in 7%, 41%, 47%, and 5% of the trajectories, respectively. As opposed to the gas phase, ISC events in aqueous solution are much less (Figure 10b and SI Figure S11). The majority of the trajectories undergo internal conversion to the S1 (77%) or S0 (13%) states. After 250 fs decay, the remaining trajectories are in the S2 (1%), S3 (1%), T1 (3%), T3 (3%), or T4 (2%) states. No ISC occurred from the S4 state. While there were 12 ISCs from the S4 state in the gas phase, the reduced initial population of the S4 state significantly lowers the probability of ISC from the S4 state with a QM/MM aqueous solution. A transition from the S3 to T6 state was observed four times. For trajectories in the S2 state, a transition to the T5 state was recorded four times, while one transition to the T6 state and one to the T4 state were present, as well. Last, three crossings from the S1 to T5 state and three reverse crossings from the T5 to S1 state happened, involving six different trajectories. All deactivated systems reached the ground state via the S1 state. Internal conversion processes are less frequent for the trajectories in aqueous solution, and the overall ISC kinetics is slower (SI Figure S8). The electronic state characters are the same for the QM/MM system as in the gas phase calculations, and trajectories eventually evolved into the *n* → π* electronic state characters for both the singlet and triplet energetically lowest excited states, whereby some S1 populated trajectories hop to the S0 state via CIs between the *n* → π* and ground electronic states.

Similarly to the gas phase results, a steep initial increase of the N–O and mutual parallel C_H_–C_H_ bond length as well as a reduced O_1_–N–O_2_ angle in the S1, T1, and T2 states are present. The distributions of the bond lengths are generally more narrow as compared to the gas phase (SI Figure S9), and no nitrophenolate is formed. The standard deviation on the average nitro C–C–N–O torsional angle steadily increases with time, reflecting a greater flexibility for out-of-plane rotation. These geometric constraints explain the reduced ISC. In the gas phase, the C–N bond lengths are typically increased at ISC points, but these extreme values are rarely reached in aqueous solution. The spin–orbit coupling values are analogue to gas phase values. No significant shift in the radial distribution functions is found within the simulation time.

As can be seen from Figure 10, there is a large difference in the 4-NP photodecay when the solvent is modeled with TDDFT as compared to QM/MM. The full ab initio trajectories were initiated in the S1 (12%), S2 (21%), S3 (63%), and S4 (4%) states depending on the respective bright states, which consistently had a predominant π → π* (HOMO → LUMO) character. Hence, as compared to QM/MM, the initial population of the S2 state was reduced in favor of the S3 state. However, it should be noted that almost all initial S3 and S4 states include a contribution of the intermolecular CT character previously described for 2-NP. If the initial points are recalculated with the CAM-B3LYP functional, the intermolecular CT states are shifted higher in energy, usually starting from the S6 excitation, while the five energetically lower singlet excited electronic states are free of this contribution. Nonetheless, the CAM-B3LYP absorption spectrum is blue-shifted by ∼30 nm as compared to the experimental spectrum (SI Figure S12). The intermolecular CT character dominates the evolution in a number of excited trajectories, eventually influencing 46% of the trajectories during the simulation time. In the most dominant cases, the initial excited electronic state character completely evolves into the intermolecular CT state, which leads to direct deactivation of the excited electronic state through a cascade of trivial crossings with energetically lower excited states until reaching the S0 state, as was observed for the deactivation of the intermolecular CT S2 state in solvated 2-NP. 36% of trajectories reached the S0 state through a final CI between the intermolecular CT and ground electronic states, while only 17% had a π → π* S1 state character before reaching the CI with the S0 state. After 250 fs of simulation time, the number of trajectories in a triplet state is similar to QM/MM. Nevertheless, all have deactivated to the T1 state, whereas with QM/MM the triplet state populations were spread over the T1, T3, and T4 states (see SI Table S5). Overall, 11 ISCs took place, and 3 reverse ISCs. A transition from the S1 to T2 state and from the S3 to T5 state was detected three times, in addition to two transitions from the S3 to T6 state. A single transition from S1 to T3, from S2 to T4, and from S1 via S0 to T1 was discovered. Most ISC transitions occurred in the first 50 fs. At the end of the simulation time, 38% of the trajectories remained in the excited singlet states (almost all in the S1 state), spread among three different electronic characters: π → π* (16%), *n* → π* (15%), and the intermolecular CT state (7%). Only π → π* (6%) and intermolecular CT state (3%) characters are observed in the final points of the trajectories ending in the T1 state. The CAM-B3LYP functional also gives the same S1 character for all S0/S1 CI points and final simulation geometries, where the S1 state is of intermolecular CT character at the used level of theory.

With full periodic TDDFT, one proton transfer to the solvent from a solute OH group in the T1 π → π* state was found, forming H_3_O^+^. The evolution of all internal coordinates is very similar compared to QM/MM results, but peaks in the N–O bond lengths are less extreme (SI Figure S9). The energy scale for deformations is comparable to gas phase results. As previously observed in the QM/MM trajectories, the cage effect again leads to a reduced ISC. The evolution of the distance between the hydroxyl and nitro groups’ oxygen atoms (SI Figure S13) indicates a limited feasibility for the solute to expand as compared to the gas phase, which is also reflected in a reduced increase of the S0 energy. The success rate for ISCs (i.e., the number of energy crossings versus the number of ISCs) is 1% with full TDDFT solvation and QM/MM as compared to 6% in the gas phase. More internal conversions to the S0 state are recorded using full TDDFT as compared to QM/MM and gas phase results due to the contribution of the intermolecular CT states in the former. The full TDDFT solvated 4-NP has an additional nonradiative relaxation pathway via the intermolecular CT state to S0. In all systems, the destabilization of the ground electronic states by geometrical deformations lifts the S0 state energy to the higher-lying CI with the S1 state. All QM/MM S0–S1 internal conversion points feature an out-of-plane, pyramidalized nitro group, while the torsion of the hydroxyl group is limited. As indicated by the optimized CI_S0/S1_ (SI Table S4), out-of-plane rotation of the hydroxyl group can also lead toward a CI. This is observed with full TDDFT explicit solvation, both in its π → π* and CT states but not in QM/MM trajectories. Moreover, all electronic states have lower excitation energies in full TDDFT solution as compared to QM/MM and gas phase (SI Table S6), and the hydrogen bonding with the nitro group further stabilizes the ground state, making it more susceptible to geometric deformations necessary to reach the CI (Figure 9c). In the intermolecular CT state, the extension of the water’s O–H bond additionally lifts the S0 state energy. Analogue to the QM/MM trajectories, no significant changes in the radial distribution functions of the nitro and hydroxyl group atoms during the full TDDFT trajectories are found within the simulation time.

## 4 Conclusions

Nitrophenols are omnipresent, toxic pollutants causing photochemical smog.^25,30^ In this study, the initial decay of 2-NP and 4-NP was simulated in the gas phase using a periodic QM/MM solvation model with electrostatic embedding and in full periodic (TD)DFT aqueous solution. To the best of our knowledge, this is the first study using SH with TDDFT and explicit periodic solvation that compares the SH including ISC in explicit solvation for both computational approaches. Whereas Xu et al. included only one singlet and two triplet excited states in their SH studies of gas phase 2-NP,^15^ the higher-lying states added in this study (two singlet and four triplet excited states) took part in the deactivation as well. ESIPT to form the aci-nitro tautomer is frequent in the gas phase. ISC is reduced in an aqueous solution, and ESIPT is limited within the 250 fs simulation time. Hence, a barrier for ESIPT is established in aqueous solution as opposed to the gas phase, resulting in reduced ISC.

Whereas a gas phase picosecond-time scale SH study of 2-NP has been published,^15^ previous 4-NP photodeactivation studies were experimental. The decay of 4-NP is energetically more complex compared to 2-NP due to the close-lying singlet excited states. Four singlet and seven triplet excited states were included, all of which were populated during the decay. Within 250 fs, 58% of the gas phase 4-NP trajectories showed ISC. In solution, this is firmly reduced due to the cage effect restricting the bond length variations.

Experimentally, the excited state dynamics of nitrophenols was found to significantly depend on the solvent and corresponding potential proton transfer processes.^17^ Ernst et al. did not clearly observe a solvent effect for 2-NP, but they acknowledge the limited experimental resolution and noise from excited state absorption.^16^ Nevertheless, previous theoretical studies included the aqueous environment via implicit solvation models or QM/MM.^14^ Bistafa et al. already concluded that a QM description of the polarized water molecules around the *para*-nitrophenolate is necessary.^19^ A significant difference between QM/MM results, with electrostatic embedding and full TDDFT calculations, was found for both 2-NP and 4-NP, indicating the significance of an accurate solvation model for the decay of nitrophenols.

With the inclusion of the water molecules at the same (TD)DFT level as the solute, additional excited electronic states are incorporated into the NAMD, for nitrophenols more specifically, the intermolecular CT states. The applied hybrid B3LYP functional correctly describes the lowest excitations and models the first peak of the experimental absorption spectrum well but introduces intermolecular CT state contributions into energetically higher excited electronic states. These contributions further increase with the excitation energies and dominate higher excitations. The CT contributions are also observed using the long-range corrected CAM-B3LYP functional, but they are shifted higher in energy. In addition, the CAM-B3LYP functional is capable of reproducing the increased absorption at lower wavelengths, even though the energies are noticeably blue-shifted.

The maxima of the radial distribution functions and in the absorption spectra are shifted with QM/MM compared to full TDDFT results. Moreover, while the QM/MM 2-NP NAMD trajectories overestimate the ISC due to the facilitated ESIPT, the QM/MM 4-NP NAMD trajectories overestimate the S1 lifetime, as CI geometries with an out-of-plane rotation of the hydroxyl group are not reached in combination with systematic lower excitation energies of the states. On the other hand, the full TDDFT solvated 4-NP excited state lifetime is potentially underestimated by the nonradiative decay mechanism involving the intermolecular CT state. The same intermolecular CT state cannot be modeled at the QM/MM level without including water molecules at the same QM level as the solute. It should be noted that the intermolecular CT states are reproducible with the CAM-B3LYP functional. The same deactivation mechanism involving a cascade of trivial crossings that eventually leads to the CI between the CT state and the ground electronic state is found with this long-range corrected functional. One proton transfer from 4-NP to the aqueous solvent was observed using full, periodic TDDFT.

For a similar push–pull molecule, *para*-nitroaniline, a strong dependence of the intersystem coupling efficiency on the solvent polarity was found.^118^ Hence, further studies with diverse solvents, longer simulation times, and other types of QM/MM coupling could give deeper insights into these widespread environmentally damaging molecules.

## Supporting Information Available

Additional figures for all compounds and tables summarizing excitation characters, excitation energies, structural parameters, and final state occupations (PDF)
