# Supplementary material for: The Role of Aqueous Solvation on the Intersystem Crossing of Nitrophenols
Source: J Chem Theory Comput. 2024 Apr 12;20(8):3258–72. doi: 10.1021/acs.jctc.3c01400 (PMC11044273; doi:10.1021/acs.jctc.3c01400)
Supplement: Supplementary file 1 — ct3c01400_si_001.pdf [file ct3c01400_si_001.pdf]

Supporting Information for:

The Role of Aqueous Solvation on the Intersystem Crossing of Nitrophenols

Eva Vandaele, Momir Mališ, and Sandra Luber*

Department of Chemistry, University of Zürich, Winterthurerstrasse 190, 8057 Zürich, Switzerland

E-mail: sandra.luber@chem.uzh.ch

S1 *ortho*-nitrophenol

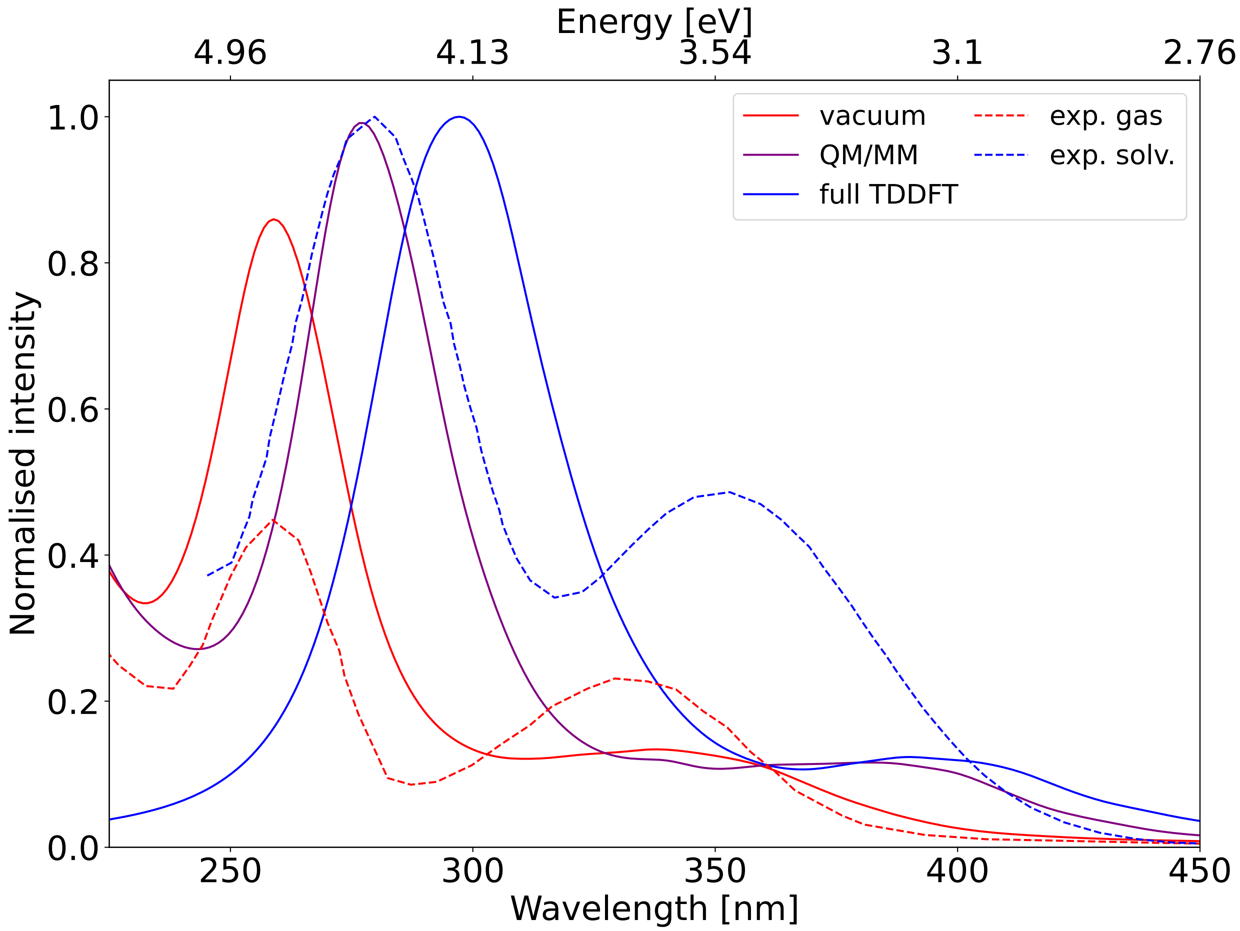

Figure S1: The TDDFT absorption spectrum of 2-NP in the gas phase (red), and in aqueous solution modelled with QM/MM (purple) and full TDDFT (blue) computed from the first 10 excited states. The dashed curves correspond to experimentally measured absorption spectra of 2-NP in the gas phase and the aqueous solution reproduced from ref.[1] and ref.,[2] respectively.

Table S1: Indication of the 2-NP TDDFT excitation characters at the S0 optimised geometry.

	Transition
S1	HOMO → LUMO (92%)
S2	HOMO−2 → LUMO (95%)
T1	HOMO → LUMO (97%)
T2	HOMO−4 → LUMO (73%)
T3	HOMO−2 → LUMO (89%)
T4	HOMO−1 → LUMO (64%)

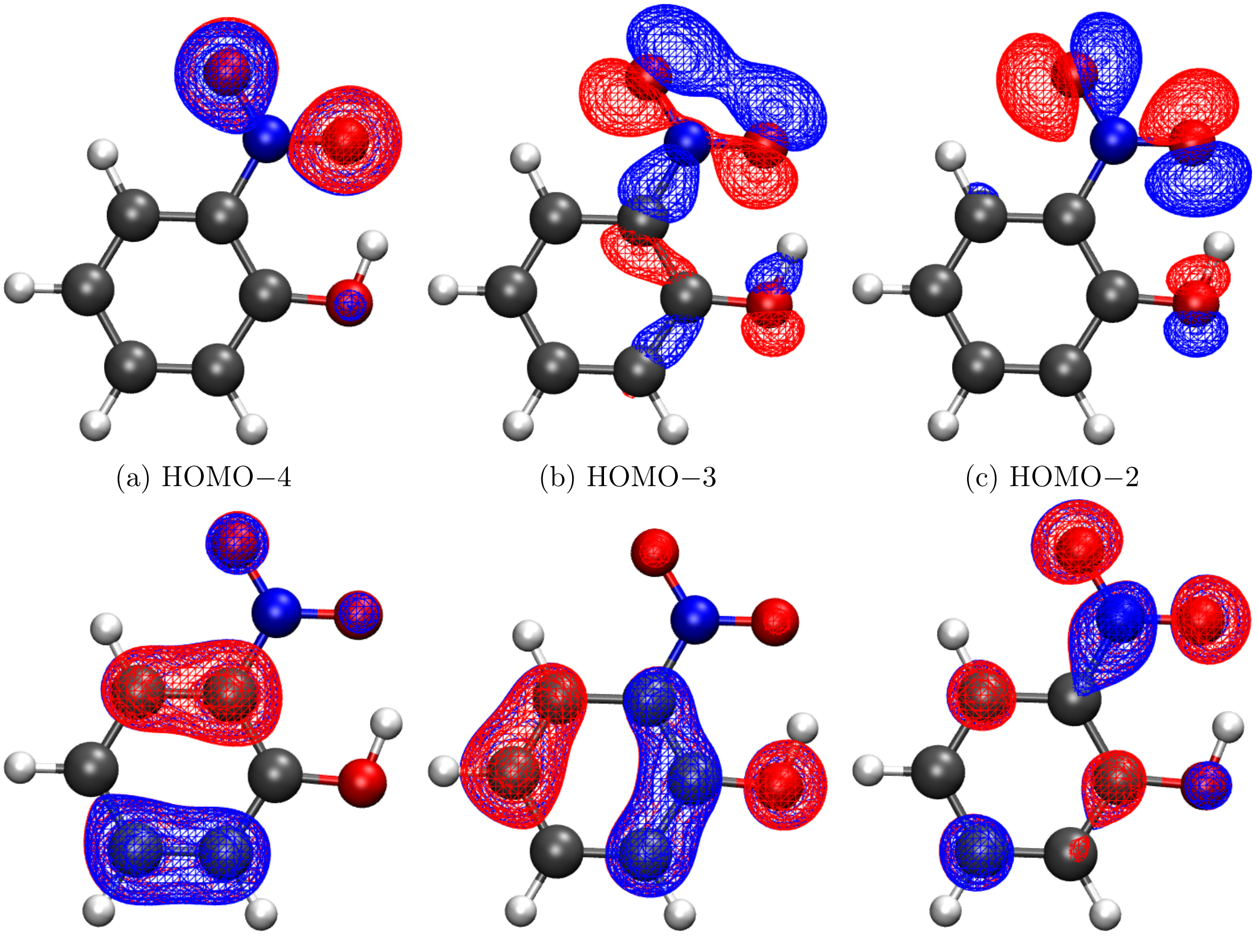

(a) HOMO−4 (b) HOMO−3 (c) HOMO−2

(d) HOMO−1 (e) HOMO (f) LUMO

Figure S2: Canonical MOs of 2-NP at the S0 optimised geometry.

Table S2: Comparison of the 2-NP TDDFT excitation energies (in eV) averaged over all initial conformations.

	gas phase	QM/MM	full TDDFT
S1	3.58 ± 0.21	3.24 ± 0.16	3.03 ± 0.23
S2	3.98 ± 0.27	3.70 ± 0.19	3.60 ± 0.21
T1	2.74 ± 0.22	2.45 ± 0.18	2.27 ± 0.23
T2	3.30 ± 0.24	3.02 ± 0.16	2.88 ± 0.18
T3	3.47 ± 0.29	3.18 ± 0.18	3.13 ± 0.19
T4	3.67 ± 0.23	3.38 ± 0.16	3.29 ± 0.18

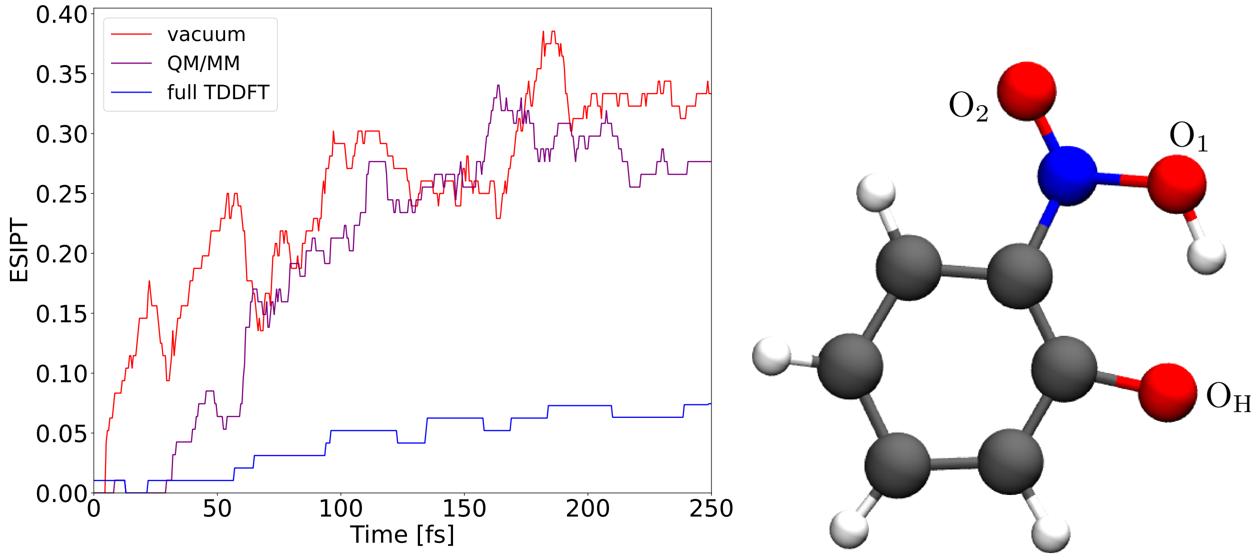

(a) (b)

Figure S3: (a) Evolution of ESIPT in the gas phase (red), QM/MM aqueous solution (purple) and full TDDFT (blue) 2-NP trajectories, whereby ESIPT was counted if the O_1–H bond length was greater than the O_H–H bond length. In the graph 0 indicates no proton transfer and 1 denotes only nitronic acid tautomers. (b) Example of a geometry at 250 fs after excitation during which ESIPT was observed.

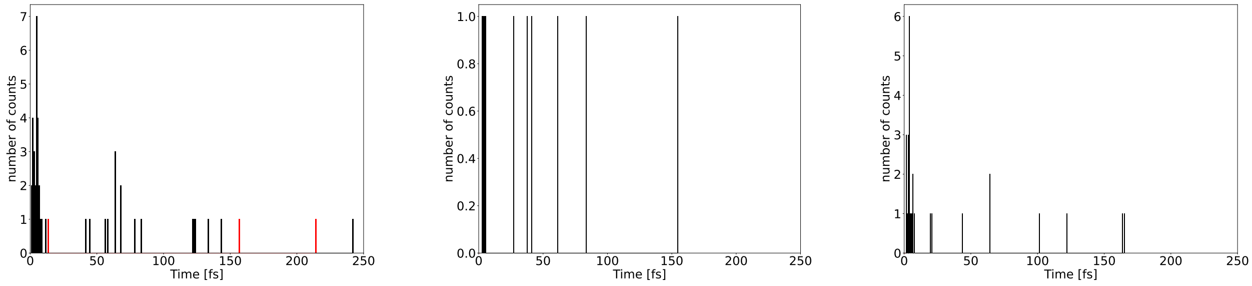

(a) (b) (c)

Figure S4: Histograms showing the timings of the intersystem crossings (black) and reverse intersystem crossings (red) in the 2-NP gas phase (a), full TDDFT solvation (b), and QM/MM (c) trajectories.

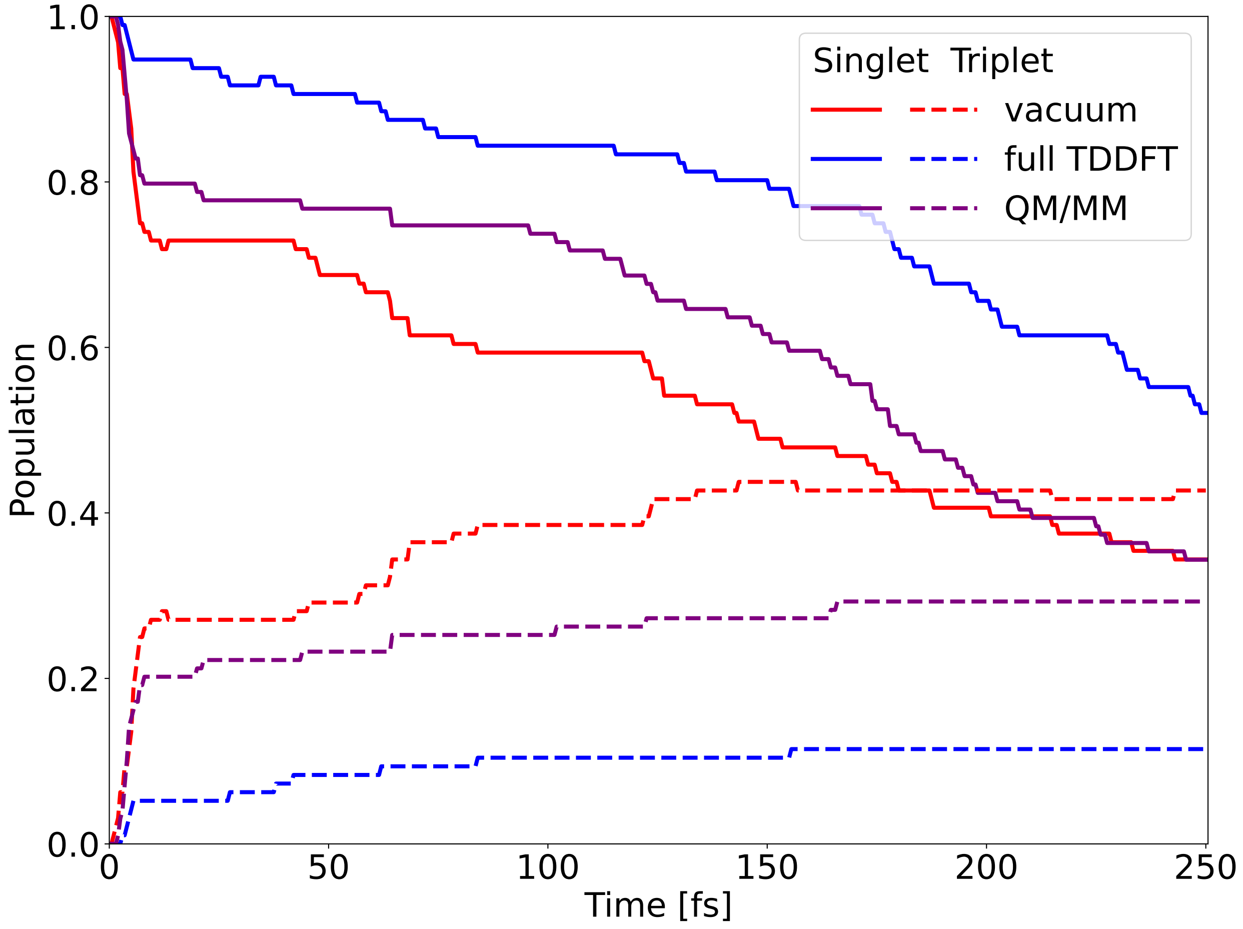

Figure S5: Cumulative evolution of all singlet (full lines) and triplet (dashed lines) electronic states for the 2-NP in the gas phase (red), full TDDFT solvation (blue), and QM/MM (purple) trajectories.

S2 *para*-nitrophenol

Table S3: Indication of the 4-NP TDDFT excitation characters at the S0 optimised geometry.

	Transition
S1	HOMO−2 → LUMO (97%)
S2	HOMO−4 → LUMO (97%)
S3	HOMO → LUMO (75%)
S4	HOMO−1 → LUMO (67%)
T1	HOMO−3 → LUMO (80%)
T2	HOMO → LUMO (88%)
T3	HOMO−2 → LUMO (94%)
T4	HOMO−4 → LUMO (96%)
T5	HOMO−1 → LUMO (82%)
T6	HOMO−1 → LUMO+1 (63%)
T7	HOMO → LUMO+1 (68%)

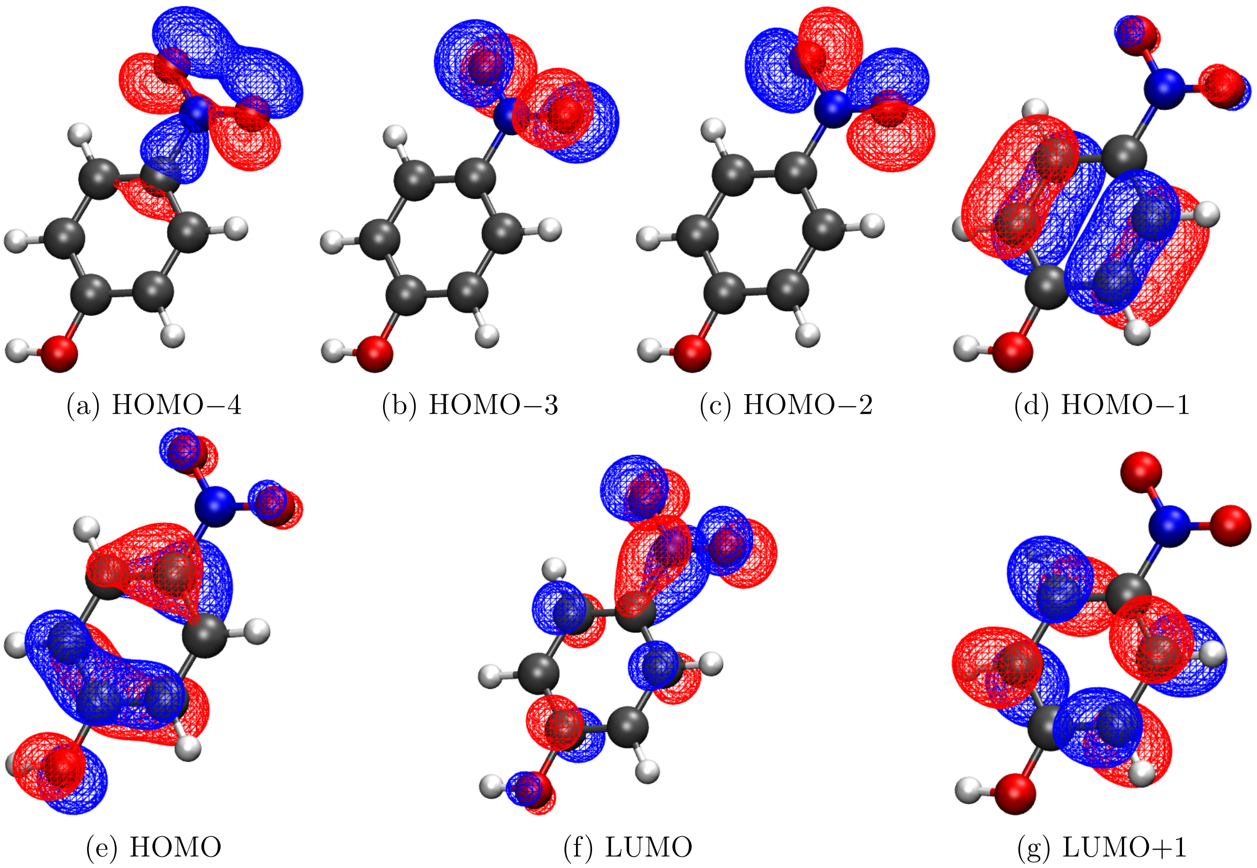

Figure S6: Canonical MOs of 4-NP at the S0 optimised geometry.

Table S4: Comparison of the 4-NP structural parameters at the S0 optimised geometry.

	$S0_{opt}$	ref. [3]	$S1_{opt}$	$T1_{opt}$	CI_{S0S1}
C–O [Å]	1.37	1.36	1.37	1.37	1.37
O–H [Å]	0.97	/	0.97	0.97	0.97
C–N [Å]	1.47	1.45	1.37	1.40	1.37
N–O [Å]	1.24	1.24	1.31	1.31	1.33
ONO [°]	124	123	106	106	90
CNO [°]	118	119	127	124	130
COH [°]	110	/	110	110	108
CCNO [°]	−179	/	−179	−167	−164
CCOH [°]	0	/	1	0	25

Table S5: Overview of the 4-NP occupations 250 fs after excitation.

	gas phase	QM/MM	full TDDFT
S0	21%	13%	54%
S1	30%	77%	36%
S2	0%	1%	1%
S3	1%	1%	0%
S4	0%	0%	0%
T1	30%	3%	9%
T2	12%	0%	0%
T3	5%	3%	0%
T4	0%	2%	0%
T5	1%	0%	0%
T6	0%	0%	0%
T7	0%	0%	0%

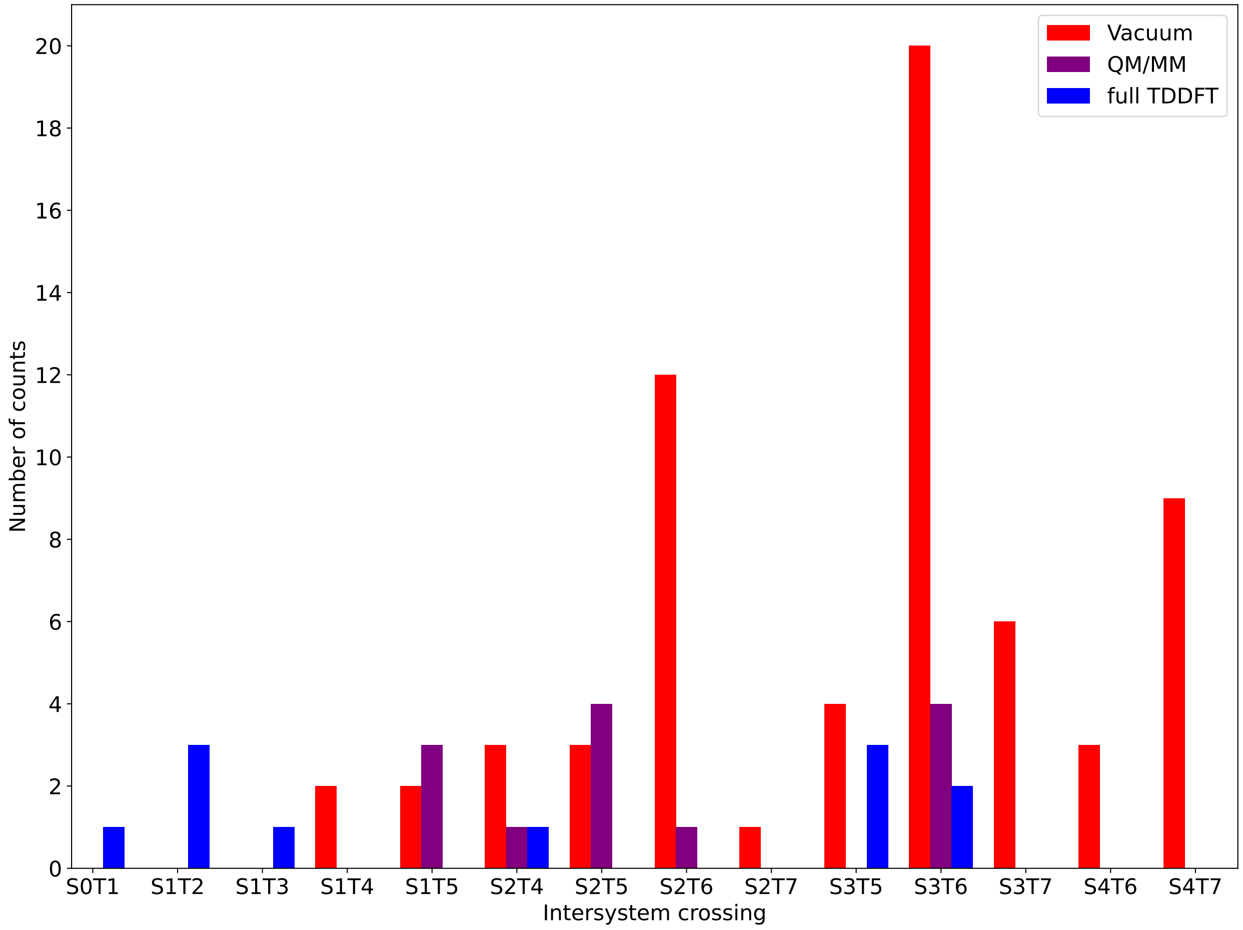

Figure S7: Number of intersystem crossings in the 4-NP SH trajectories grouped per interacting electronic state pairs.

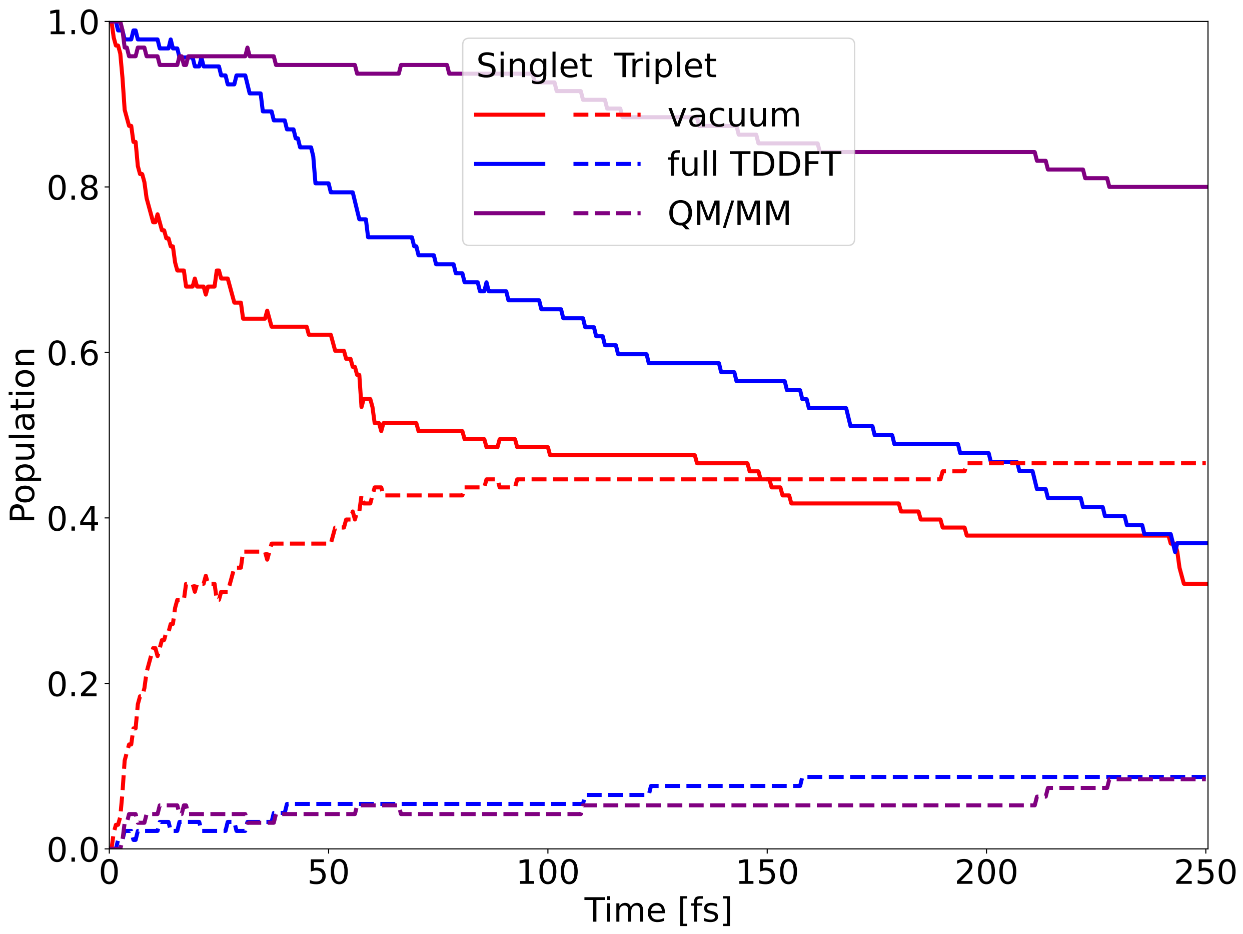

Figure S8: Cumulative evolution of all singlet (full lines) and triplet (dashed lines) electronic states for the 4-NP in the gas phase (red), full TDDFT solvation (blue), and QM/MM (purple) trajectories.

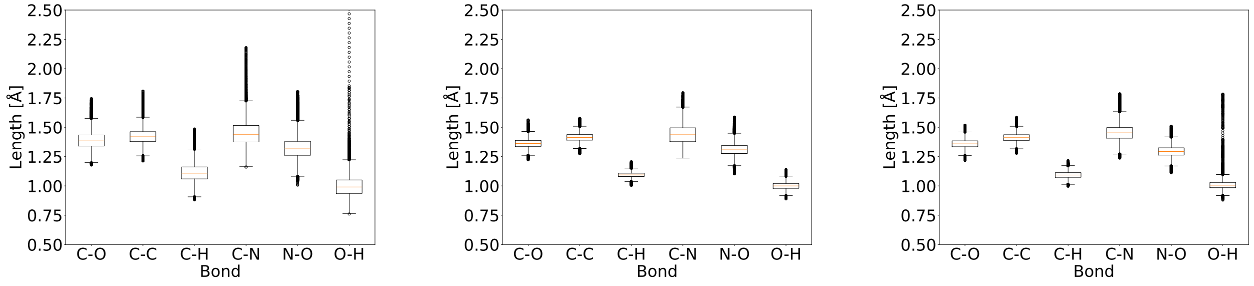

(a) (b) (c)

Figure S9: Boxplots showing the bond lengths of the 4-NP gas phase (a), QM/MM (b), and full TDDFT solvation (c) trajectories. The outliers in the O–H bond lengths are caused by the formation of a *para*-nitrophenolate anion. An analogue narrowing of the distributions after solvation in water is observed for all angles and torsional angles.

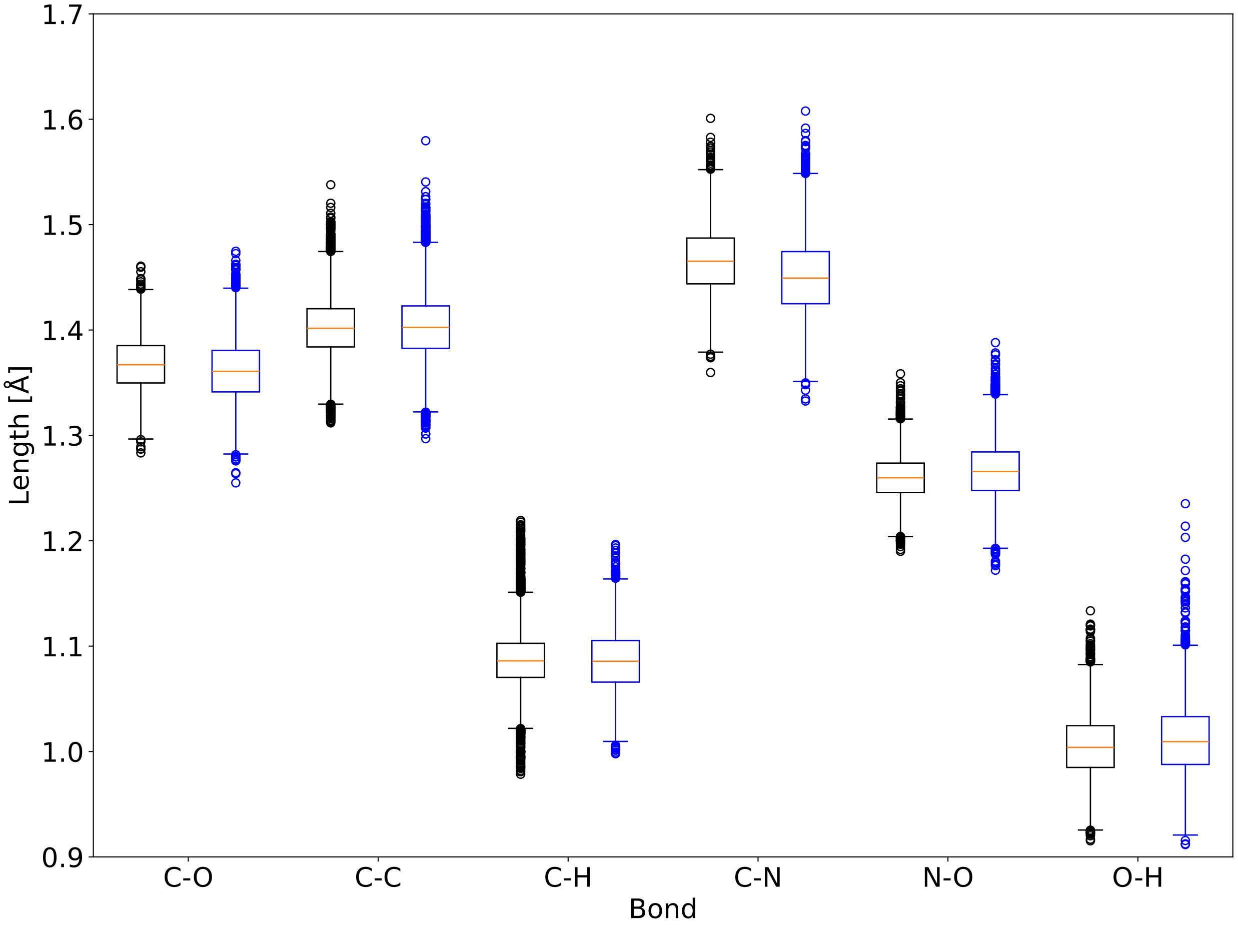

Figure S10: Boxplots showing the bond lengths of the 4-NP QM/MM (black), and full DFT solvation (blue) S0 NVT trajectories.

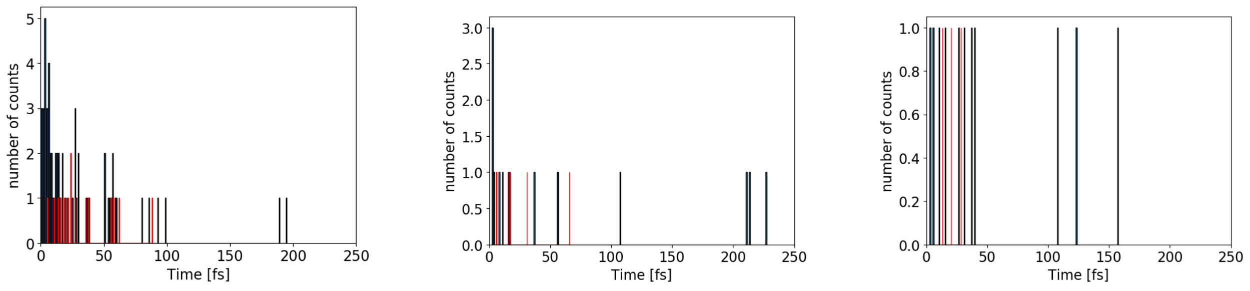

(a) (b) (c)

Figure S11: Histograms showing the timings of the intersystem crossings (black) and reverse intersystem crossings (red) in the 4-NP gas phase (a), QM/MM (b), and full TDDFT solvation (c) trajectories.

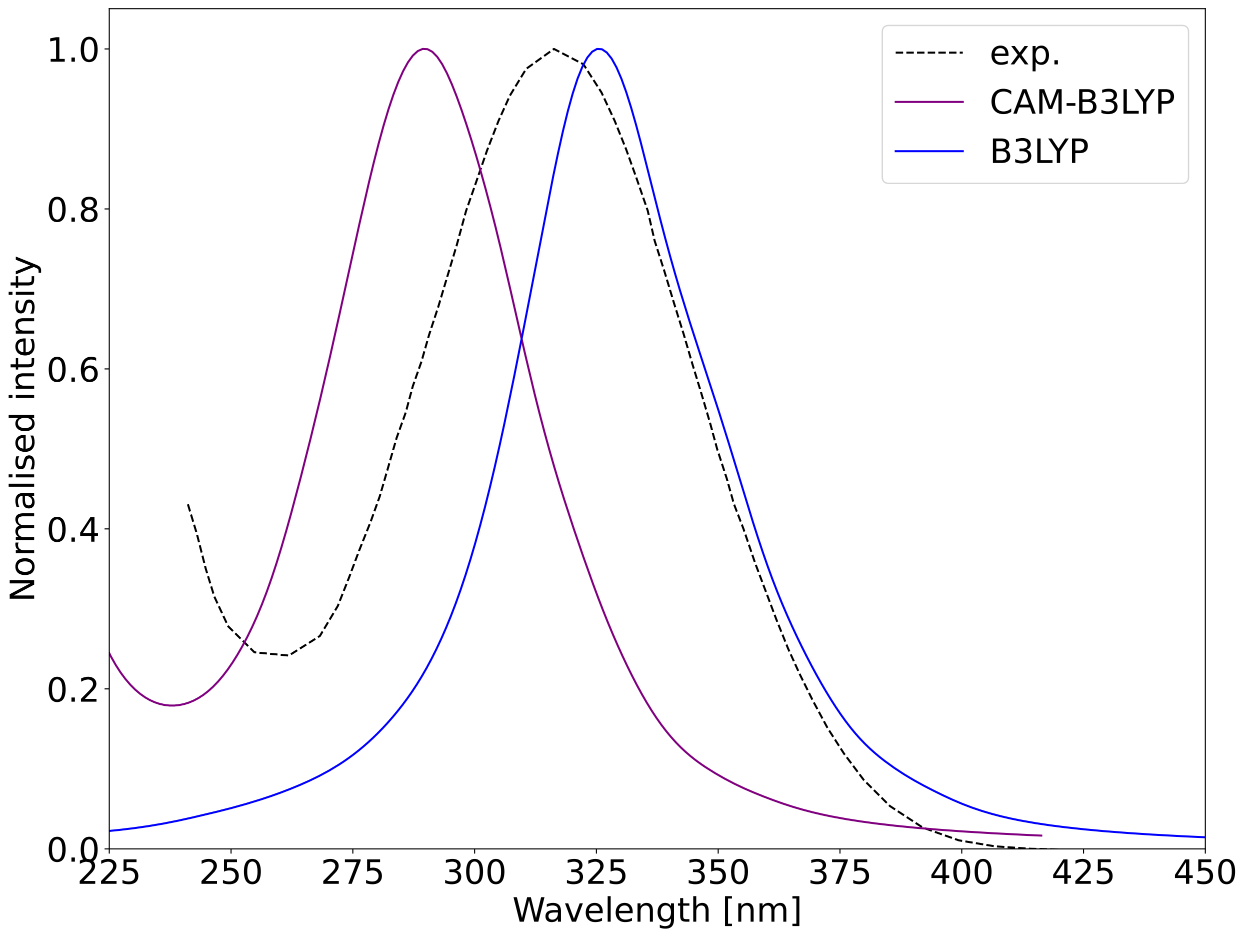

Figure S12: Calculated absorption spectra for the full TDDFT solvated 4-NP with the CAM-B3LYP (purple line) and B3LYP (blue line) functionals and compared to the experimental spectrum of aqueous 4-NP solution (dashed black line).[2]

Table S6: Comparison of the 4-NP TDDFT excitation energies (in eV) averaged over all initial conformations.

	gas phase	QM/MM	full TDDFT
S1	3.84 ± 0.37	3.61 ± 0.16	3.47 ± 0.15
S2	4.37 ± 0.20	4.02 ± 0.13	3.68 ± 0.16
S3	4.52 ± 0.17	4.14 ± 0.11	3.87 ± 0.12
S4	4.70 ± 0.17	4.32 ± 0.11	4.14 ± 0.17
T1	3.08 ± 0.26	2.71 ± 0.12	2.39 ± 0.18
T2	3.32 ± 0.35	3.06 ± 0.14	3.00 ± 0.16
T3	3.48 ± 0.29	3.21 ± 0.17	3.18 ± 0.18
T4	3.90 ± 0.20	3.61 ± 0.13	3.35 ± 0.16
T5	4.09 ± 0.18	3.84 ± 0.11	3.84 ± 0.13
T6	4.34 ± 0.19	4.24 ± 0.13	4.09 ± 0.15
T7	4.58 ± 0.21	4.40 ± 0.12	4.25 ± 0.16

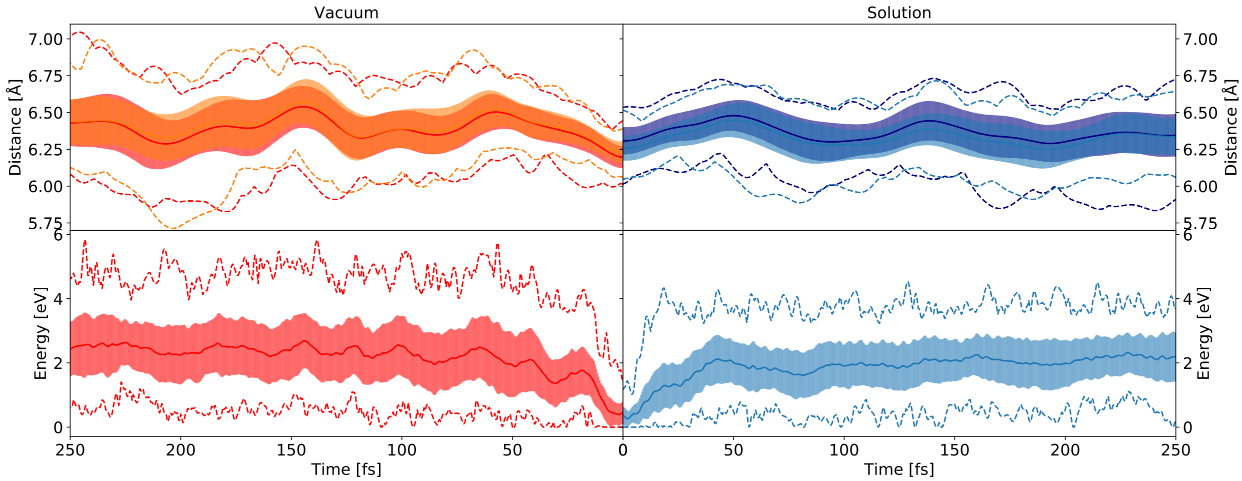

Figure S13: Top: evolution of the average distance [Å] between the 4-NP hydroxyl oxygen and nitro oxygens in the gas phase (left) and full TDDFT aqueous solution (right). Bottom: evolution of the average ground state energy [eV] during the SH trajectories. All energy values are relative to the minimum energy value of each trajectory set. The coloured bands indicate the standard deviation, while the dotted lines show the minimum and maximum values.

References

(1) Ernst, H. A.; Wolf, T. J.; Schalk, O.; González-García, N.; Boguslavskiy, A. E.; Stolow, A.; Olzmann, M.; Unterreiner, A. N. Ultrafast Dynamics of o-Nitrophenol: An Experimental and Theoretical Study. *J. Phys. Chem. A* **2015**, *119*, 9225–9235.

(2) Bailey-Darland, S.; Krueger, T. D.; Fang, C. Ultrafast Spectroscopies of Nitrophenols and Nitrophenolates in Solution: From Electronic Dynamics and Vibrational Structures to Photochemical and Environmental Implications. *Molecules* **2023**, *28*, 601.

(3) Ando, R. A.; Borin, A. C.; Santos, P. S. Saturation of the electron-withdrawing capability of the NO_2 group in nitroaromatic anions: Spectroscopic and quantum-chemical evidence. *J. Phys. Chem. A* **2007**, *111*, 7194–7199.
